# ADNP dysregulates methylation and mitochondrial gene expression in the cerebellum of a Helsmoortel–Van der Aa syndrome autopsy case

**DOI:** 10.1186/s40478-024-01743-w

**Published:** 2024-04-18

**Authors:** Claudio D’Incal, Anke Van Dijck, Joe Ibrahim, Kevin De Man, Lina Bastini, Anthony Konings, Ellen Elinck, Claudia Theys, Illana Gozes, Zlatko Marusic, Mirna Anicic, Jurica Vukovic, Nathalie Van der Aa, Ligia Mateiu, Wim Vanden Berghe, R. Frank Kooy

**Affiliations:** 1 Department of Medical Genetics, University of Antwerp, Prins Boudewijnlaan 43/6, 2650 Edegem, Antwerp Belgium; 2 Protein Chemistry, Proteomics and Epigenetic Signaling (PPES), Epigenetic Signaling lab (PPES), Department of Biomedical Sciences, University of Antwerp, Universiteitsplein 1, 2610 Wilrijk, Antwerp Belgium; 3 Family Medicine and Population Health (FAMPOP), Department of Medicine and Health Sciences, University of Antwerp, Antwerp, Belgium; 4 The Elton Laboratory for Molecular Neuroendocrinology, Department of Human Molecular Genetics and Biochemistry, Faculty of Medical & Health Sciences, Adams Super Center for Brain Studies and Sagol School of Neuroscience, Tel Aviv University, Tel Aviv, Israel; 5 Clinical Department of Pathology and Cytology, University Hospital Centre Zagreb, Zagreb, Croatia; 6 Division of Pediatric Gastroenterology, Hepatology and Nutrition, Department of Pediatrics, School of Medicine, University Hospital Centre Zagreb, Zagreb, Croatia

**Keywords:** Activity-dependent neuroprotective protein (ADNP), Methylation, Chromatin remodeler, Autophagy, Mitochondria, Post-mortem brain, Helsmoortel–Van der Aa syndrome, Sirtuin 1 (SIRT1)

## Abstract

**Background:**

Helsmoortel–Van der Aa syndrome is a neurodevelopmental disorder in which patients present with autism, intellectual disability, and frequent extra-neurological features such as feeding and gastrointestinal problems, visual impairments, and cardiac abnormalities. All patients exhibit heterozygous de novo nonsense or frameshift stop mutations in the *Activity-Dependent Neuroprotective Protein* (*ADNP*) gene, accounting for a prevalence of 0.2% of all autism cases worldwide. ADNP fulfills an essential chromatin remodeling function during brain development. In this study, we investigated the cerebellum of a died 6-year-old male patient with the c.1676dupA/p.His559Glnfs*3 *ADNP* mutation.

**Results:**

The clinical presentation of the patient was representative of the Helsmoortel–Van der Aa syndrome. During his lifespan, he underwent two liver transplantations after which the child died because of multiple organ failure. An autopsy was performed, and various tissue samples were taken for further analysis. We performed a molecular characterization of the cerebellum, a brain region involved in motor coordination, known for its highest ADNP expression and compared it to an age-matched control subject. Importantly, epigenome-wide analysis of the ADNP cerebellum identified CpG methylation differences and expression of multiple pathways causing neurodevelopmental delay. Interestingly, transcription factor motif enrichment analysis of differentially methylated genes showed that the *ADNP* binding motif was the most significantly enriched. RNA sequencing of the autopsy brain further identified downregulation of the WNT signaling pathway and autophagy defects as possible causes of neurodevelopmental delay. Ultimately, label-free quantification mass spectrometry identified differentially expressed proteins involved in mitochondrial stress and sirtuin signaling pathways amongst others. Protein–protein interaction analysis further revealed a network including chromatin remodelers (ADNP, SMARCC2, HDAC2 and YY1), autophagy-related proteins (LAMP1, BECN1 and LC3) as well as a key histone deacetylating enzyme SIRT1, involved in mitochondrial energy metabolism. The protein interaction of ADNP with SIRT1 was further biochemically validated through the microtubule-end binding proteins EB1/EB3 by direct co-immunoprecipitation in mouse cerebellum, suggesting important mito-epigenetic crosstalk between chromatin remodeling and mitochondrial energy metabolism linked to autophagy stress responses. This is further supported by mitochondrial activity assays and stainings in patient-derived fibroblasts which suggest mitochondrial dysfunctions in the ADNP deficient human brain.

**Conclusion:**

This study forms the baseline clinical and molecular characterization of an ADNP autopsy cerebellum, providing novel insights in the disease mechanisms of the Helsmoortel–Van der Aa syndrome. By combining multi-omic and biochemical approaches, we identified a novel SIRT1-EB1/EB3-ADNP protein complex which may contribute to autophagic flux alterations and impaired mitochondrial metabolism in the Helsmoortel–Van der Aa syndrome and holds promise as a new therapeutic target.

**Graphical abstract:**

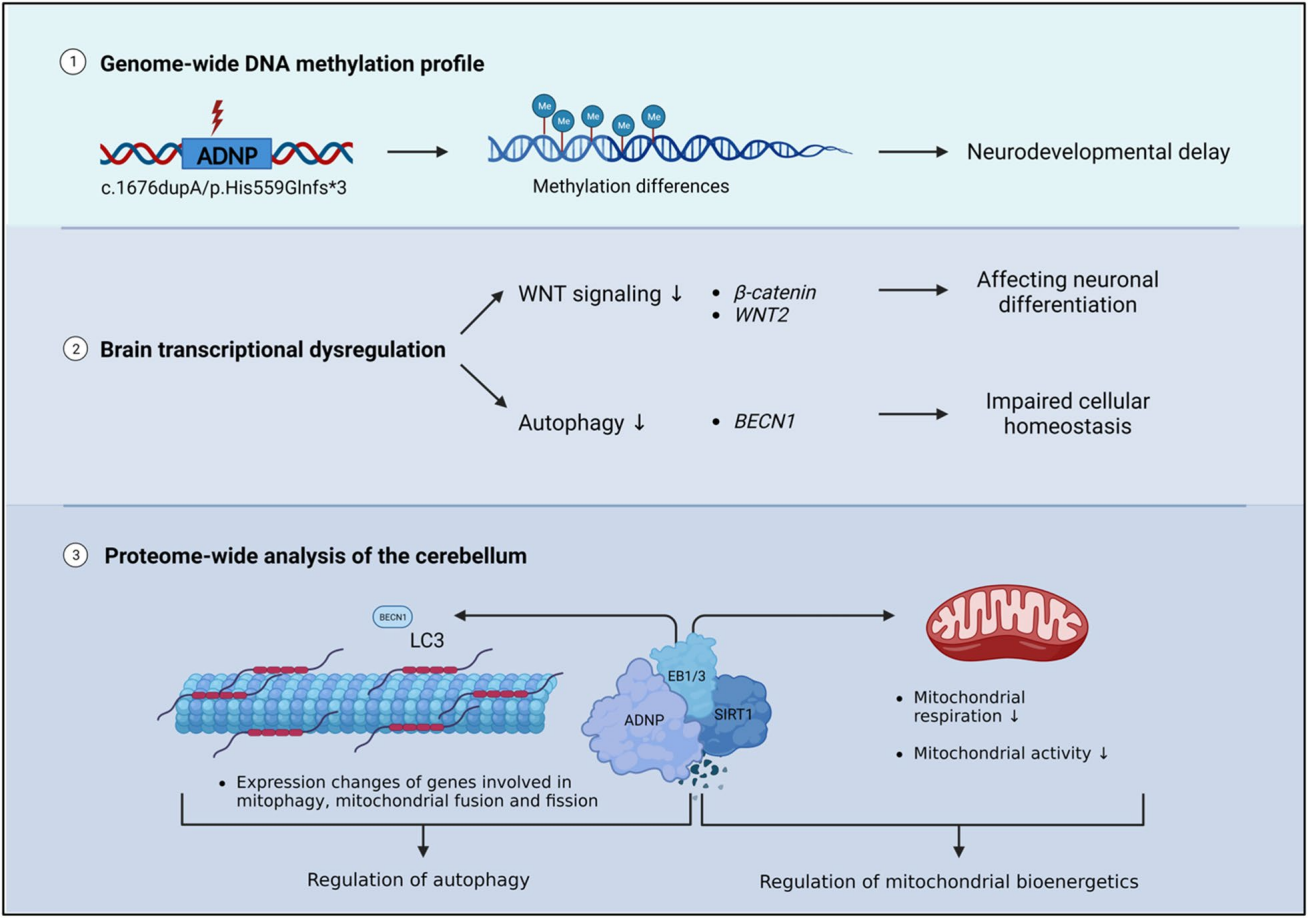

**Supplementary Information:**

The online version contains supplementary material available at 10.1186/s40478-024-01743-w.

## Highlights

- The *ADNP* patient mutation affects genome-wide methylation and leads to neurodevelopmental abnormalities.
- The ADNP brain transcriptome reveals impaired neuronal differentiation and cellular homeostasis by aberrant signaling of the WNT pathway and autophagy process.
- ADNP forms a complex with SIRT1 through the microtubule end-binding proteins EB1 and EB3.
- Mitochondrial gene expression is impaired in the ADNP brain and patient-derived cellular models.

**Supplementary Information:**

The online version contains supplementary material available at 10.1186/s40478-024-01743-w.

## Introduction

The development of whole exome sequencing (WES) has substantially increased our insights in the genetic causes of neurodevelopmental disorders by detection of de novo mutations by comparing the exome of the proband to that of its parents [1]. Using this method, mutations in the *Activity Dependent-Neuroprotective Homeobox Protein* (*ADNP*) gene have been discovered, contributing to a neurogenetic syndrome called Helsmoortel–Van der Aa syndrome (*OMIM 615873*), with a prevalence of 0.2% of global autism cases [2, 3]. Patients show a clinical presentation of mild to severe intellectual disability (ID), autism spectrum disorder (ASD), global developmental delay (GDD), motor and speech delay, behavior abnormalities and deficiencies in several organ systems such as gastrointestinal problems [4]. In recent screening studies, *ADNP* appears one of the most frequently mutated genes with a hundred percent disease penetrance [5, 6].

While massive screening studies now have cumulated in the discovery of over a thousand genes that are involved in ID and/or ASD, our molecular and functional understanding of the pathophysiology of these genes is lagging far behind. For instance, despite a wealth of information, many biochemical aspects of the function of ADNP in the brain remain unknown [7]. Spanning a genomic length of almost 40 kb, the longest transcript of the *ADNP* gene contains six exons of which only the last three translate to the actual protein [8]. Functional domains of ADNP include nine zinc fingers, a bipartite nuclear localization signal (NLS), a homeobox domain with ARKS motif, a heterochromatin protein 1 (HP1)-interacting PxVxL motif, and the neuroprotective octapeptide sequence NAPVSIPQ (NAP) [8–12]. In the nucleus, ADNP plays a role in chromatin remodeling: it binds directly to other chromatin remodelers, including the BAF complex members BRG1, ARID1A, and SMARCC2 by its C-terminal tail as demonstrated in a HEK293 human embryonic kidney cell line [9], and to CHD4 by its N-terminus as well as HP1β by its C-terminus in the repressive ChAHP complex discovered in murine embryonic stem cells, where it competes with CTCF for a common set of binding motifs [13]. Besides, a stable triplex of ADNP, BRG1 and CHD4 was also reported in murine stem cells [14], while POGZ and HP1γ form a nuclear complex with ADNP in the embryonic mouse cortex [15]. Most recently, ADNP was predicted to interact with the WRD5-SIRT1-BRG1-HDAC2 including YY1 complex [16]. Although the involvement of ADNP in chromatin remodeling functions has been firmly established, the role of these protein complexes in the human brain remains to be determined. In terms of function, ADNP is involved in neuronal tube closure and brain development, controlling expression of hundreds if not thousands of genes [17].

The chromatin function of ADNP is reflected by specific, aberrant methylation patterns in the blood of patients. In fact and almost unique to ADNP, two partially opposing methylation patterns have been described, depending on the location of the mutation. Whereas mutations located at the 3′-end and 5′-end of the *ADNP* gene (outside of nucleotides c.2000–2340) represent a Class I episignature with a pattern of overall hypomethylated CpGs, mutations in the central region (within nucleotides 2000–2340) of the gene show rather CpG hypermethylation [18, 19]. Interestingly, the hypermethylated region, encompassing the recurrent p.Tyr719* *ADNP* mutation, is associated with a more severe clinical presentation [4, 20]. Cytoplasmic roles for ADNP have also been suggested e.g., involvement in autophagy by binding LC3 [21], and interactions with the cytoskeleton via the microtubule end-binding proteins (EB1/EB3) [22, 23], with *Adnp* deficiency resulting in impaired axonal transport and impaired dendritic spines [23–26]. Additionally, ADNP interacts with other cytoskeletal proteins such as SHANK3 and actin as well as with the armadillo sequence of beta-catenin, important for WNT signaling [14, 27, 28].

However, none of the above-mentioned studies have been performed in disease-relevant tissue. Instead, immortalized human cell lines, murine tissues, embryonic stem cells or other model systems have been investigated. Here, we present a unique case study on autopsy material of a six-year-old child with the heterozygous c.1676dupA/p.His559Glnfs*3 de novo* ADNP* mutation*.* By combining in-depth epigenetic, transcriptomic, and proteomic studies in the cerebellum of this post-mortem ADNP subject, we were able to confirm the involvement of pathways such as the WNT-signaling in the Helsmoortel–Van der Aa syndrome as well as to demonstrate ADNP involvement in autophagy and mitochondrial (dys)function(s).

## Materials and methods

### Post-mortem tissues and subjects

Clinical information of a nine-year-old female patient was obtained under informed written consent from the Institute Born-Bunge vzw IBB NeuroBioBank of the University of Antwerp and transferred with written informed consent under HMTA20210040 after approval of the Ethics Committee of the Antwerp University Hospital/University of Antwerp. The female subject, used as a control in this study, showed symptoms analogous to a sporadic form of Rett syndrome and died following obstructive apnea. Twelve hours after death, cerebellar tissue was collected during the autopsy and frozen in liquid nitrogen or fixed in formaldehyde. Frozen section, paraffin sections, and celloidin embeddings were extensively investigated by an expert pathologist, resulting in no morphological abnormalities in all brain regions, except some fibrillar gliosis in the hippocampus. Comparisons to other sections of an age-matched control showed similar cytology and no neuronal loss. The substantia nigra contained normal amounts of melanin granules and the cerebellum contained no loss of Purkinje cells. Clinical information of a dead six-year-old male patient with the heterozygous c.1676dupA/p.His559Glnfs*3 *ADNP* mutation was received under informed consent under B300201627322 and approved by the Ethics Committee of the Antwerp University Hospital. The patient died because of multiple organ failure. Cerebellar tissue was collected during autopsy following a 35-h post-mortem interval, subsequently frozen in liquid nitrogen or fixed in formaldehyde. Clinical evaluation was performed by at least one expert clinical geneticist. The *ADNP* mutation was confirmed by Sanger sequencing using the forward primer 3′-TGATGTGCAAGTGCATCAGA-5′ and reverse primer 3′-TGTGCACTTCGAAAAAGAACAT-5′. Conservation of the amino acids changed by the *ADNP* mutation was verified using ClustalW.

### Plasmid constructs and site-directed mutagenesis

The pCMV3 expression vector encoding human wild-type ADNP fused to either an N-terminal GFPSpark® or N-DYKDDDDK (Flag®) tag was purchased from Sino Biological (*HG11106-ANG*; *HG11106-NF*). The c.1676duplA mutation was introduced in the N-DYKDDDDK (Flag®) *ADNP* expression vector by PCR mutagenesis using the Q5® Site-Directed mutagenesis kit (New England Biolabs; *E0554S*) according to manufacturer’s protocol. Mutagenesis primers were designed using the NEBaseChanger Tool (http://nebasechanger.neb.com/). The mutation was inserted with the forward primer: 5′-ACACTAACATCCATCTCCTG-3’ and the reverse primer: 5′-TGACTACCCTGCTGCAAT-3′ by thermocycling with an annealing temperature of 60 °C. DNA was purified from transformed high-efficiency NEB 5-alpha competent *E. coli* cells using the NucleoSpin Plasmid EasyPure Mini kit (Macherey Nagel; *740727.50*) according to the manual. The mutation was confirmed by Sanger sequencing.

### Cell lines and culture conditions

HEK293T cells (ATCC; *CRL-3216™*) were cultured at low passage number in DMEM (Gibco™; *11965092*), supplemented with 10% fetal bovine serum (Gibco™; *26140079*) and 1% penicillin/streptomycin (Gibco™; *15070063*). Age- and sex matched Epstein-Barr virus transformed lymphoblastoid cell lines (LCLs) of healthy subjects (n = 4) and patients with different *ADNP* mutations (n = 6) (Additional file 1: Table S1) were cultured in RPMI (Gibco™; A1049101), supplemented with 15% fetal bovine serum (Gibco™; 26140079), 1% penicillin/streptomycin (Gibco™; 15070063), 1% sodium pyruvate (Gibco™; 11360070), and 1% GlutaMAX (Gibco™; 35050061). Age- and sex matched skin fibroblasts of two unrelated asymptomatic subjects and two patients with different *ADNP* mutations (n = 2) (Additional file 1: Table S1) were cultured in RPMI (Gibco™; A1049101), supplemented with 15% fetal bovine serum (Gibco™; 26140079), 1% penicillin/streptomycin (Gibco™; 15070063), 1% sodium pyruvate (Gibco™; 11360070), and 1% GlutaMAX (Gibco™; 35050061). Human primary cell lines were obtained from consenting individuals, guardians, tending clinicians, or parents. All procedures were carried out following the guidelines and regulations of the University of Antwerp/University Hospital of Antwerp (UZA) and approved by the Ethics Committee of the Antwerp University Hospital. All cell lines were cultured in a humidified incubator at 37%O_2_/5%CO_2_.

### AlfaFold 3D-structural protein modeling

The predicted 3D-structure of human wild-type ADNP (Uniprot; Q9H2P0) was acquired using the AlfaFold Protein Structure Database (https://alphafold.ebi.ac.uk/). The p.His559Glnfs*3 mutant was queried in the amino acid sequence of wild-type ADNP. Wild-type and mutant ADNP proteins were modeled using AlfaFold2 with ColabFold (https://colab.research.google.com/github/sokrypton/ColabFold/blob/main/AlphaFold2.ipynb), an online integrating AlfaFold2 pipeline for protein structure modeling combined with many-against-many sequence searching (MMSeqs2), and HHSearch. ChimeraX (UCSF, version 1.5) was used for visualization and annotation of the structural ADNP protein domains using the generated PBD output file as input.

### Cellular ADNP transfection system

HEK293T were transiently transfected with 5 µg of human expression vectors: (1) wild-type ADNP with an N-terminal GFPSpark®-tag, (2) wild-type ADNP with an N-terminal DYKDDDDK (Flag®)-tag, or (3) mutant c.1676dupA ADNP fused to an N-terminal DYKDDDDK (Flag®)-tag using Lipofectamine™ 3000 Transfection Reagent (Invitrogen; *L3000008*) in accordance with the manufacturer’s protocol. Co-transfections were performed with equal amounts of both wild-type and mutant ADNP expression vectors. Transfection efficiency was about 70% in line with the manufacturer’s tested performance. Cells were harvested after 24-h for subcellular protein fractionation followed by western blotting.

### ADNP expression analysis: total protein extraction and subcellular protein fractionation

After transient transfection of the *ADNP* expression vector of interest, HEK293T cells were detached with TrypLE™ Express Enzyme (1X), phenol red (Gibco™; *12605028*), subsequently washed with ice-cold DPBS (Gibco™; *14040133*). Cerebellar tissue obtained from the post-mortem control subject and died ADNP patient was homogenized with the TissueRuptor II (Qiagen; *9002755*) with mixing at the lowest speed. For total protein extraction, cells and tissue were lysed in ice cold RIPA buffer (150 mM NaCl, 50 mM Tris, 0.5% sodium deoxycholate, 1% NP-40 and 2% sodium dodecyl sulfate), supplemented with the cOmplete™, Mini, EDTA-free Protease Inhibitor Cocktail (Roche; *04693159001*) together with PhosSTOP™ phosphatase inhibitor (Roche; 4906845001). Lysis occurred for 15 min at 4 °C with agitation and cell debris was removed by centrifuging 15 min at maximal speed in a precooled centrifuge. For subcellular fractionation of transfected HEK293T cells, a final amount of 10 × 10^6^ cells was lysed and gradually separated in cytoplasmic, membrane, nuclear soluble, chromatin-bound, and cytoskeletal protein extracts using the Subcellular Protein Fractionation Kit for Cultured Cells (Thermo Scientific™; *78840*) following the manufacturer’s instructions. The protein concentration was estimated with the Pierce™ BCA Protein Assay Kit (Thermo Scientific™; *23225*). ADNP expression was investigated in the cytoplasmic, chromatin-bound, and cytoskeletal protein fractions.

### Immunoblotting

A total amount of 20 μg protein lysate was reduced with NuPAGE™ Sample Reducing Agent (Invitrogen; *NP0009*) in NuPAGE™ LDS Sample Buffer (Invitrogen; *NP0007*). Samples were heated for 10 min at 70 °C and subsequently loaded for separation using a Bolt™ 4 to 12%, Bis–Tris, 1.0 mm, Mini Protein Gels (Invitrogen; *NW04120BOX*) using Bolt™ MOPS SDS Running Buffer (Invitrogen; *B0001*) at 120 V. The Precision Plus Protein™ All Blue Prestained Protein Standard (Biorad; *#1610373*) was used for estimation of the molecular weight in all experiments. After separation, proteins were transblotted onto Amersham™ Protran® Premium nitrocellulose membranes (Cytiva; *GE10600008*) using a Mini Trans-Blot® cell (Biorad; *1703930*) with a transfer buffer containing 25 mM Tris, 192 mM glycine and 20% methanol (pH 8.3). Successful protein transfer was checked with a Ponceau S solution (Sigma Aldrich; *P7170*). Nitrocellulose membranes were blocked with either 5% blocking-grade non-fat dry milk (NFDM) (Carl Roth; *T145.4*) or 5% bovine serum albumin (BSA) (Carl Roth; *CP84.1*) dissolved in tris-buffered saline (TBST) for one hour at room temperature with agitation. Primary antibodies (Additional file 1: Table S2) were tested and optimized to raise the least amount of background signals. Signal amplification was achieved by incubation with an appropriate HRP-conjugated immunoglobulins (Agilent) in a 1:2000 dilution in either 5% blocking-grade non-fat dry milk/TBST or 5% BSA/TBST solution. The signal was detected using the Pierce™ ECL Western Blotting Substrate (Thermo Scientific™; *32106*). The West Femto Maximum Sensitivity Substrate (Thermo Scientific™; *34095*) was used For ADNP detection specifically [29]. Image acquisition was executed with the Amersham™ Imager 680 (Cytiva). Monoclonal GAPDH (Cell signaling technology; *4317*), Histone H3 (Abcam; *10799*), and β-actin (Sigma-Aldrich; *A5441*) (Table S3) were used as loading controls for all the experiments. Image Quantification was performed using ImageJ software. Graphical representation was performed in GraphPad Prism version 9.3.1 using an unpaired student T-test assuming equal variances and normal distribution. Full western blot images are show as supplementary materials (Additional file 2: Data S11).

### Human methylation EPIC BeadChip array and data processing

Total DNA was isolated from the cerebellar tissue of the post-mortem control subject and patient cerebellum (n = 1) using the DNeasy Blood and Tissue Kit (Qiagen; *69504*) according to the manufacturer’s instructions. Subsequently, bisulfite conversion of 250 ng isolated DNA was performed using the EZ DNA Methylation Kit (Zymo Research, D5001). To confirm successful bisulfite conversion, a methylation-conserved fragment of the human *SALL3* gene was amplified using the following primers: 5′-GCGCGAGTCGAAGTAGGGC-3′ as forward primer and 5′-ACCCAACGATACCTAATAATAAAACC-3 as reverse primer with the PyroMark PCR kit (Qiagen; *978703*). Amplified products were separated on a 1.5% agarose gel stained with GelRed® Nucleic Acid Gel Stain (Biotium; *41002*). The TrackIt™ 100 bp DNA Ladder (Invitrogen; *10488-058*) was used as a reference marker. Bisulfite-converted samples were hybridized on the Infinium Human Methylation EPIC BeadChip (Illumina; *20020531*) as described in the manufacturer’s protocol. EPIC chips will be analyzed using the Illumina Hi-Scan system, a platform integrating more than 850,000 methylation sites quantitatively across the genome at single-nucleotide resolution. Raw intensity files were first quality checked and processed using the minfi package (v 1.38.0) [30]. Signal intensities were normalized using quantile normalization and beta values were calculated. Probes with a detection *p*-value higher than 0.01 were excluded. Non-CpG probes, probes with known single nucleotide polymorphisms (SNPs), multihit probes and probes on the X-/Y-chromosomes were filtered out. Probe annotation was carried out using the Illumina Infinium MethylationEPIC v1.0 B5 manifest file. All annotations (i.e., CpG islands, shelve, and shore regions) are reported based on the GRCh37/hg19 human genome build. We calculated the difference in methylation of the signals acquired in patient versus control subject with a focus on CpG probes showing over 20% methylation, i.e., hypomethylation (Δβ-values < −0.2) and hypermethylation (Δβ-value > 0.2). We determined gene ontology enrichment using the Metascape webtool [31]. Protein–protein network interactions of ADNP with the identified hypermethylated and hypomethylated genes were predicted using the STRING database version 12.0. The iRegulon plugin in Cytoscape was used to detect the transcription factors, their targets, and the motifs/tracks associated with co-expression of the hypomethylated and hypermethylated genes.

### Targeted pyrosequencing analysis

Biologically-relevant genes exhibiting a methylation difference of Δβ > 0.2 (hypermethylation) and Δβ < −0.2 (hypomethylation) between patient and control cerebellum were selected for pyrosequencing validation. Briefly, the required primers (i.e., forward, reverse, and sequencing primers) were designed using the PyroMaker Assay Design 2.0 software (Qiagen) according to the manufacturer’s instructions (Additional file 1: Table S3). Bisulfite-converted DNA fragments were PCR amplified using the PyroMark PCR kit (Qiagen; *978703*). Successful PCR amplification was assessed by tris-boric acid-EDTA (TBE) electrophoresis at 1.5% agarose gel, after which the PyroMark Q24 Instrument (Qiagen) was used to perform pyrosequencing. Biotinylated PCR products were immobilized on streptavidin-coated Sepharose beads (GE Healthcare; *17511301*), captured by the PyroMark vacuum Q24 workstation, washed and denatured. Single-stranded PCR products were subsequently released into a 24-well plate and annealed to the sequencing primer for 5 min at 80 °C. After completion of the pyrosequencing run, results were analyzed using the PyroMark Q24 software (Qiagen). Graphical representation was performed with GraphPad Prism version 9.3.1.

### Total RNA extraction and sequencing of post-mortem brains and ADNP lymphoblastoid cell lines

Total RNA was extracted from the cerebellum of the control subject and the patient with the c.1676duplA/p.His559Gln*3 *ADNP* mutation (n = 1) as well as from control and patient LCLs with different *ADNP* mutations (n = 4 controls, n = 6 patients) using the RNeasy Mini Kit (Qiagen; *74106*) according to the manufacturer’s protocol. RNA concentration was determined with the Qubit™ RNA Broad Range Assay Kit (Invitrogen™; *Q10211*) and the 260/280 ratio, indicative of RNA purity, was checked using NanoDrop™ 2000/2000c Spectrophotometer (Thermo Scientific™; *ND-2000*). RNA integrity was verified with Agilent RNA Screentape Assay on the 2200 TapeStation instrument (Agilent; *G2964AA*). Samples with the highest RIN score (RIN > 6.5) were selected and sent to Novogene for RNA sequencing (RNAseq) (Additional file 1: Table S1). All sequencing data was mapped to the human annotated genome GRCh38.p13 (Ensembl v106) with STAR, after adapter removal and reads cleaning with trimmomatic. Gene expression quantification was performed with featureCounts (subread package). We calculated gene expression differences in our post-mortem brains using NOISeq (R package), a non-parametric method for one-versus-one cases that reports the log2-ratio of the two conditions (M) and the value of the difference between conditions (D). A gene is considered to be differentially expressed if its corresponding M and D values are likely to be higher than in noise. A similar analysis was performed for the functional enrichment exploration for the up- and downregulated genes found by NOISeq at q > 0.95. Differential gene expression analysis for the LCL samples was performed with the DESeq2, an R package. The genes having a BH-adjusted FDR, *p* value < 0.05, and an absolute value of log2FC >  = 0.5 were considered biologically relevant and further analyzed for functional enrichment (clusterProfiler R package with fGSEA function for the gene set enrichment analysis and enrichGO for overrepresentation analysis in GO ontologies and KEGG pathways). Additional data visualization was supported by BigOmics, a user-friendly and interactive cloud computing based bioinformatics platform for the in-depth analysis, visualization, and interpretation of transcriptomics data [32]. Ultimately, we performed a meta-analysis of the differentially expressed genes identified in the LCLs and post-mortem brains based on gene ID intersection and looked for conserved ADNP-relevant genes beyond their tissue-specific expression (brains versus LCLs).

### RT-PCR gene expression analysis

RT-PCR was used to confirm a selection of genes from the RNA sequencing experiment (LCLs, post-mortem brains and common genes between data sets) by converting 1 µg of total extracted RNA to cDNA using the SuperScript™ III Reverse Transcriptase kit (Invitrogen™; *18080093*). Primer efficiencies were optimized using a standard dilution curve method on pooled cDNA samples from controls and patients per dataset (90% > E > 110%). RT-PCR was performed in triplicate using the CFX384 Touch Real-Time PCR Detection System (BioRad; *1855484*) with primers listed in Additional file 1: Table S4 using the Takyon™ No ROX SYBR 2X MasterMix (Eurogentec; *UF-NSMT-B0701*). Reference gene stability was assessed using the geNorm method in qbase + (Biogazelle), after which were selected for normalization. Data analysis was performed in qbase + (Biogazelle) with a maximum deviation of 0.5 per triplicate using the stable housekeeping genes *ACTB*, *B2M*, and *UBC*. Statistical analysis was performed in GraphPad Prism 9.3.1 using an unpaired student T-test assuming unequal variances (post-mortem brains) and a Mann Witney U-test for unpaired measure (LCLs).

### Label-free quantification (LFQ) mass spectrometry

Cerebellar tissue obtained from the post-mortem control subject and patient was homogenized with the TissueRuptor II (Qiagen; *9002755*) with mixing at the lowest speed. Tissues were lysed and homogenized in ice cold RIPA buffer (150 mM NaCl, 50 mM Tris, 0.5% sodium deoxycholate, 1% NP-40 and 2% sodium dodecyl sulfate), supplemented with the cOmplete™, Mini, EDTA-free Protease Inhibitor Cocktail (Roche; 04693159001) together with PhosSTOP™ phosphatase inhibitor (Roche; *4906845001*). Lysis occurred for one hour at 4 °C with agitation and cell debris was removed by centrifuging 30 min at maximal speed in a precooled centrifuge. The protein concentration was estimated with the Pierce™ BCA Protein Assay Kit (ThermoScientific™; *23225*). Protein reduction, alkylation and digestion were performed with the ProteoSpin™ On-Column Proteolytic Digestion Kit (Norgen; *17500*) according to manufacturer’s protocol. A nano-liquid chromatography (LC) column (Dionex ULTIMATE 3000) coupled online to a Q Exactive™ Plus Hybrid Quadrupole-Orbitrap™ Mass Spectrometer (Thermo Scientific™) was used for the MS analysis. Peptides were loaded for five technical replicates onto a 75 μm × 150 mm, 2 μm fused silica C18 capillary column, and mobile phase elution was performed using buffer A (0.1% formic acid in Milli-Q water) and buffer B (0.1% formic acid in 80% acetonitrile/Milli-Q water). The peptides were eluted using a gradient from 5% buffer B to 95% buffer B over 120 min at a flow rate of 0.3 μL/min. The LC eluent was directed to an ESI source for Orbitrap analysis. The MS was set to perform data dependent acquisition in the positive ion mode for a selected mass range of 375–2000 m/z for quantitative expression difference at the MS1 (140,000 resolution) level followed by peptide backbone fragmentation with normalized collision energy of 28 eV, and identification at the MS2 level (17,500 resolution). The *.RAW files were exported and processed in PEAKS AB 2.0 (Bioinformatics Solutions Inc.). The files were searched using target-decoy matching using the human UniProt database, with the false discovery rate set at 1%. Trypsin was indicated as the enzyme and up to two miscleavages were allowed. Carbamidomethylation was set as a fixed modification. Label-Free Quantification (LFQ) and Match Between Runs were used using default settings. PEAKS intensities were uploaded in MetaboAnalyst5.0, subsequently quantile normalized, log-transformed and autoscaled. An unpaired student T-test was used to compare the LFQ intensities between groups and those with *p*-values ≤ 0.05 were considered significant. The protein IDs with significant values were subjected to Ingenuity Pathway Analysis (IPA) and the String Database to identify affected canonical pathways and functional protein–protein interaction network. A selection of differentially expressed proteins was ultimately confirmed with immunoblotting as described above.

### Animals

Male C57BL/6JCr wild-type mice were purchased from Charles River at the age of 10 weeks with a body weight of 25 g. Animals were socially housed with a maximum of eight animals in standard mouse cages (22.5 cm × 16.7 cm × 14 cm) at constant humidity and temperature in a 12/12 h light–dark cycle. Food and water were available ad libitum. Cage enrichment was supplied by a platform, tunnel, and extra cotton sticks. Ex vivo experiments, such as immunohistochemistry (IHC) and co-immunoprecipitation (CoIP), were performed with cerebellar tissue at the age of 10 weeks. All conducted experiments were in compliance with the EU Directive 20,120/63/EU under ECD code 2022–59 after approval by the Animal Ethics Committee of the University of Antwerp.

### Immunohistochemistry of frozen murine brain sections (IHC-Fr)

Male C57BL/6JCr wild-type mice were used for immunohistochemistry experiments at the age of 10 weeks. All animals were anesthetized by an intraperitoneal injection of 133.3 mg/kg Dolethal (Vetoquinol; *BE-V171692*), then transcardially perfused for four minutes with 0.1 M phosphate-buffered saline (PBS), subsequently for six minutes with ROTI®Histofix 4% paraformaldehyde solution (pH 7) (Carl Roth; *3105*) using steady perfusion rate of 12 rpm (2 ml/min). Whole brains were removed from the skull and cut in half along the midline. The two hemispheres were placed in ROTI®Histofix 4% paraformaldehyde (pH 7) (Carl Roth; *3105*) for two hours at room temperature, washed in PBS (0.01 M; pH 7.4) and transferred in 20% sucrose/PBS for overnight incubation at 4 °C. Tissue samples were embedded in PELCO® Cryo-Embedding Compound (Ted Pella, Inc.; *27300*) and stored at − 80C. Tissue was cut in sections of approximately 10 µm thickness using the Leica CM1950 Cryostat Microtome (Leica Biosystems, Wetzlar, Germany) and transferred to VWR® SuperFrost® Plus, Adhesion Slides (VWR; *631-0108*). The sections were washed three times using PBS. After blocking and permeabilization with PBS containing 0.05% thimerosal, 0.01% NaN_3_, 0.1% BSA, 1% Triton X-100 and 10% normal horse serum, sections were incubated overnight with primary ADNP antibody (Abcam; *ab300114*) or SIRT1 (Abcam; *ab189494*) antibody 1:500 diluted in the blocking/permeabilization buffer at room temperature. Tissue sections were washed six times with PBS, followed by a 4-h incubation with Cy3-conjugated Fab Fragment donkey anti-rabbit (Jackson ImmnoResearch Europe Ltd; *711-167-003*) antibody 1:2000 diluted in PBS containing 0.05% thimerosal, 0.01% NaN3, 0.1% BSA and 10% normal horse serum. After six final washing steps in PBS, nuclei were stained with 5 µg/ml DAPI for 5 min, followed by three washes in PBS. Immunostained cryosections were mounted in Citifluor™ AF1 Mountant Solution (Electron Microscopy Sciences; *17,970–100*). Confocal images were obtained using a Leica SP8 confocal scanning microscope (Leica-microsystems, Wetzlar, Germany) equipped with a 405-nm diode laser (to detect DAPI) and a white light laser (WLL) used at 555 nm to visualize Cy3. Images were acquired with a 20 × objective (HC PL APO 20x/0.75 IMM CORR CS2). Acquired images were analyzed in FIJI image analysis freeware [33]. Nuclei were identified as DAPI + regions after automated thresholding of the smoothed DAPI channel (gaussion blur with kernel size 2).

### Co-immunoprecipitation (Co-IP) assay

Proteins were extracted from the wild-type mouse cerebellum using N-PER™ Neuronal Protein Extraction Reagent (Thermo Scientific; *87,792*), supplemented with 1 mg NAP/Davunetide (MedChemExpress; *HY-105066*) to enhance EB1/EB3 binding [22, 23], and subjected to Co-IP analysis with the Pierce™ Co-Immunoprecipitation Kit (Thermo Scientific™; *26149*) according to the manufacturer’s protocol. Briefly, 10 μg of antibodies of interest EB1 (Abcam; *ab53358*) and EB3 (Abcam; *ab157217*) were cross-linked to 50 μl of AminoLink Plus Coupling Resin. An amount of 1 mg of protein lysate was incubated overnight at 4 °C on an end-over-end shaker (VWR; *444-0503*). Protein elution was performed in three steps: 10 μL, 35 μl, and 50 μl respectively. The immunoprecipitated materials were subsequently investigated by immunoblotting using the following primary antibodies (Additional file 1: Table S2): rat monoclonal EB1 (Abcam; *ab53358*), rabbit monoclonal EB3 (Abcam; *ab157217*), rabbit monoclonal ADNP antibody (Abcam; *ab300114*) and SIRT1 (Abcam; *ab189494*). In addition, Pierce™ Control Agarose Resin (crosslinked 4% beaded agarose) was used as negative control (IgG). Upon immunoblotting (see above) proteins were visualized using the SuperSignal™ West Femto Maximum Sensitivity Substrate (ThermoScientific™; *34094*) after labeling with the appropriate secondary antibody (Agilent) in a 1:2000 dilution.

### Motif analysis and molecular docking of ADNP and SIRT1 to microtubule-end binding proteins 1 and 3 (EB1/3)

Motif analysis of murine Adnp (UniProt; Q9Z103), Sirt1 (UniProt; Q53Z05), Eb1 (UniProt; Q61166), and Eb3 (UniProt; Q6PER3) was performed using Eukaryotic Linear Motif (ELM) (http://elm.eu.org). Three-dimensional models were either generated with AlfaFold (https://alphafold.ebi.ac.uk) or obtained from the AlfaFold database (see above) and used to predict protein–protein interactions between Adnp and Sirt1 with both Eb1 and Eb3 using the ClusPro server (ClusPro2.0)141-144 (https://cluspro.org), a widely-used protein–protein docking tool. The top 10 resulting motifs were superimposed in UCFS ChimeraX (version 1.6.1.) to present the most probable binding interaction with Adnp and Sirt1.

### Screening RNA sequencing data using a mitophagy gene panel

Mitophagy-related gene signature was obtained by clustering analysis of RNA sequencing data from the ADNP brain autopsy and LCLs of ADNP patients and control lines upon gene set enrichment analysis with a customized gene toolbox [34] in the Omics playground v2.8.22 (Additional file 1: Table S5). We confirmed the expression of mitophagy- and mitochondrial-related genes (Additional file 1: Table S4) using RT-PCR as described above.

### Autophagy flux assessment

The autophagy flux was determined in ADNP patient and control LCLs by treatment with 160 nM of bafilomycin A1 (Santa Cruz Biotechnology, sc-2021550) for 2 h. Untreated cells and bafilomycin A1-treated cells were collected by centrifugation, and subjected to western blotting as described above. We detected expression of autophagy markers anti-p62/SQSTM1 (Abcam; *ab56416*) with an 1:2000 dilution and anti-LC3 (Abcam; *ab192890*) with an 1:1000 dilution in untreated and treated conditions to assess the autophagy flux. All western blots were controlled by GAPDH incubation (Cell signaling technology; *4317*). Image Quantification was performed using ImageJ software. Graphical representation was performed in GraphPad Prism version 9.3.1 using a 2-way ANOVA with Šídák's multiple comparisons test.

### Live cell imaging: mitochondrial redox state and subcellular localization

Intact fibroblasts of control and ADNP patients (n = 2) were seeded at a density of 4 × 10^6^ cells in a 6-well plate for live cell imaging and subsequently stained with 250 nM fluorescent probe MitoTracker® Red CM-H2XRos (Invitrogen; *M7513*) according to the manufacturer’s protocol to assess mitochondrial redox state and subcellular localization. The redox state of mitochondria is determined by the levels of NAD^+^/NADH, FAD/FADH2, NADP^+^/NADPH, glutathione/glutathione disulfide (GSH/GSSG) and reactive oxygen species (ROS), which reflect mitochondrial metabolic activity and overall fitness. If the electron transport chain is compromised or there is an imbalance in the redox state, leading to increased ROS production. When CM-H2XRos enters the mitochondria, they are oxidized depending on the relative amount of reactive oxygen species (ROS) present in the mitochondria. The oxidation leads to a change in the fluorescence properties of the red dye. The emitted red fluorescence signal was measured with a multimode microplate reader (Tecan Spark™) and analyzed using the Spark Control™ V3.2 application. The fluorescent signal was statistically quantified using an unpaired Student T-test assuming equal variances in GraphPad Prism 9.3.1. Additionally, fibroblasts were imaged with the Olympus CKX53 fluorescence microscope (Olympus, Antwerp, Belgium) to visualize subcellular localization of the mitochondria with better resolution.

### Determination of mitochondrial DNA copy number

Total DNA was isolated from ADNP LCLs and skin fibroblasts as compared to their controls using the DNeasy Blood and Tissue Kit (Qiagen; *69504*) according to the manufacturer’s instructions. Mitochondrial DNA copy number (mtDNA-CN) was determined using RT-PCR. Briefly,the cycle threshold (Ct) value of a mitochondrial-specific (*tRNAleu*) and nuclear-specific (*B2M*) target were determined in triplicate for each sample using the following primers: tRNA_LEU_-Fwd: 5′-CACCCAAGAACAGGGTTTGT-3′ and tRNA_LEU_-Rev: 5′-TGGCCATGGGTATGTTGTTA-3′ and B2M-Fwd: 5′-TGCTGTCTCCATGTTTGATGTATCT-3′ and B2M-Rev: 5′-TCTCTGCTCCCCACCTCTAGGT-3′. The difference in Ct-values (ΔCt) for each replicate represents a raw relative measure of mtDNA-CN.

### Seahorse XF cell mito stress test

ADNP patient and unrelated sex- and age-matched control fibroblasts (n = 2) were cultured on Seahorse XFp miniplates with a density of 4 × 10^4^ cells (Agilent Technologies; *103725-100*) and incubated overnight at 37% O_2_/5% CO_2_. Prior to the Seahorse XS Cell Mito Stress Test assay, the fibroblast medium was replaced with Seahorse XF RPMI medium (pH 7.4) (Agilent Technologies; *103576-100*), supplemented by 1.0 M Seahorse XF Glucose Solution (Agilent Technologies; *103577-100*), 100 mM Seahorse XF Pyruvate Solution (Agilent Technologies; *103578-100*), and 200 mM Seahorse XF L-Glutamine Solution (Agilent Technologies; *103579-100*). The drug ports of the sensor cartridge were loaded with 1 µM Oligomycin (port A), 0.7 µM Carbonyl cyanide-4- (trifluoromethoxy)phenylhydrazone (FCCP) (port B), and 0.5 µM Rotenone/Antimycin A (Rot/AA) (port C). Next, cells seeded in the Seahorse XF HS Miniplates, together with the sensor cartridge, were loaded into the Seahorse XF HS Mini Analyzer (Agilent; S7852A) and subjected to the Agilent Cell Mito Stress Test assay (Agilent; 103010-100) to determine the real-time oxygen consumption rate (OCR) for 1.5 h. First, the baseline respiration was measured (basal OCR) prior to mitochondrial perturbation by sequential injection of 1.5 µM oligomycin (a complex V inhibitor to decrease the electron flow through electron transport chain (ETC)); 3 µM FCCP (the uncoupling agent to promote maximum electron flow through ETC), and a mixture of 0.5 µM Rotenone/Antimycin A (complex I and complex II inhibitors, respectively, to shut down the mitochondria-related respiration). All compounds were included in the Seahorse XFp Cell Mito Stress Test Kit (Agilent; *103010-100*). The data was analyzed using Agilent Seahorse analytics (Agilent Seahorse Analytic). Statistical analysis was performed in GraphPad Prism 9.3.1 using an unpaired student T-test assuming equal variances.

## Results

### Clinical presentation

**Fig. 1:**
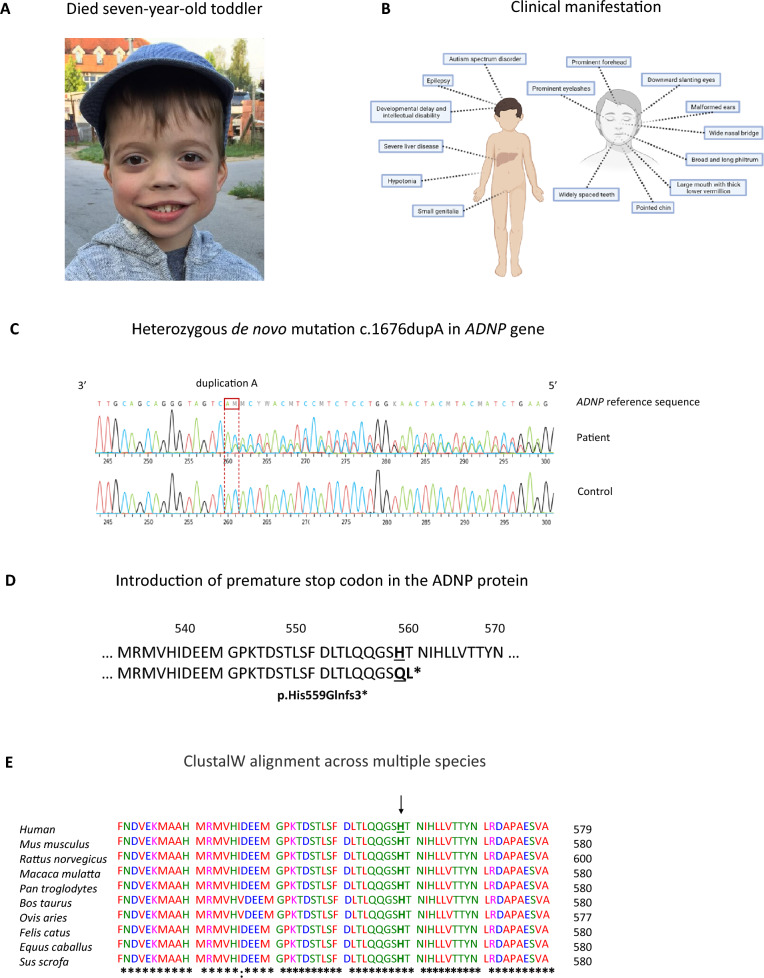
Identification of a heterozygous de novo mutation in the *ADNP* gene. (**A**) Facial photograph of the six-year-old child (https://www.adnpfoundation.org/). (**B**) Schematic representation of the clinical manifestation of the patient with Helsmoortel–Van der Aa syndrome, including autism, severe ID, and epilepsy. (**C**) DNA sequencing chromatogram of control and patient alleles, confirming a heterozygous nucleotide duplication (c.1676duplA) in the *ADNP* gene, (**D**) replacing the histidine at residue 559 with glutamic acid with a frameshift of two amino acids and introduction of a stop codon (p.His559Glnfs*3). (**E**) ClustalW alignment across multiple species of ADNP amino acids 520–580. Almost all residues of the ADNP protein are highly conserved amongst vertebrates. The arrow (↓) indicates the species-conserved histidine (H) residue, which is altered in the patient to a glutamic acid (Q) residue. The asterisk (*) indicates positions which have a single, fully conserved residue. A colon (:) indicates conservation between amino acid residues of similar properties

The patient was born prematurely, at 32 weeks of gestational age, from healthy, non-consanguineous parents. His birth weight was 1790 g, the Apgar score was 10/10. An intracranial hemorrhage grade III was diagnosed. Clinical reports showed that the patient presented with motor delays, developmental delays, autism spectrum disorder, hypotonia, and small genitalia. His parents also reported visual impairments, feeding and eating problems, as well as sleep disorders. Phenotypically, the patient presented with a prominent forehead and eyelashes, downward slanting eyes, malformed ears, wide nasal bridge, broad and long philtrum, large mouth with thick lower vermillion, pointed chin and widely spaced teeth (Fig. 1A, B), all well-defined characteristics described in a cohort of 78 Helsmoortel–Van der Aa patients [2, 4] (Additional file 1: Table S6). At the age of 2.5 years, he developed an upper respiratory tract infection complicated with hepatitis and seizures. He was transferred to ICU where supportive treatment and plasmapheresis were started. Liver biopsy showed extensive necrosis of parenchyma and moderate cholestasis. MRI showed diffuse cortical atrophy of the brain parenchyma, marked reduction in volume of white matter as well as gliosis in both frontal and temporoparietal lobes that could indicate the sequelae of acute hepatic encephalopathy. He developed refractory generalized epilepsy and received a combination treatment of antiepileptic drugs, e.g., carbamazepine, oxcarbamazepine, levetiracetam, clonazepam, clobazam and topiramate. During his lifespan, he underwent two liver transplantations and received immunosuppressants. Following the second liver transplant, at the age of six years and three months old, the child passed away because of multiple organ failure. An autopsy was performed, and various tissue samples were donated with informed consent. Molecular testing had indicated that the patient was negative for any inheritable metabolic disorders. Whole-exome sequencing (WES) of the patient’s blood revealed a heterozygous de novo duplication of adenine at position 1676 in the *ADNP* gene at position chr20:50,893,037-50,893,039 (RefSeq isoform ENST00000621696.5 Human GRCh38/hg38). The mutation was confirmed by Sanger sequencing (Fig. 1C). It converts the histidine (His) residue at position 559 to glutamic acid (Gln), leading to a frameshift mutation with a premature stop codon two amino acids downstream (Fig. 1D, E).

**Fig. 2:**
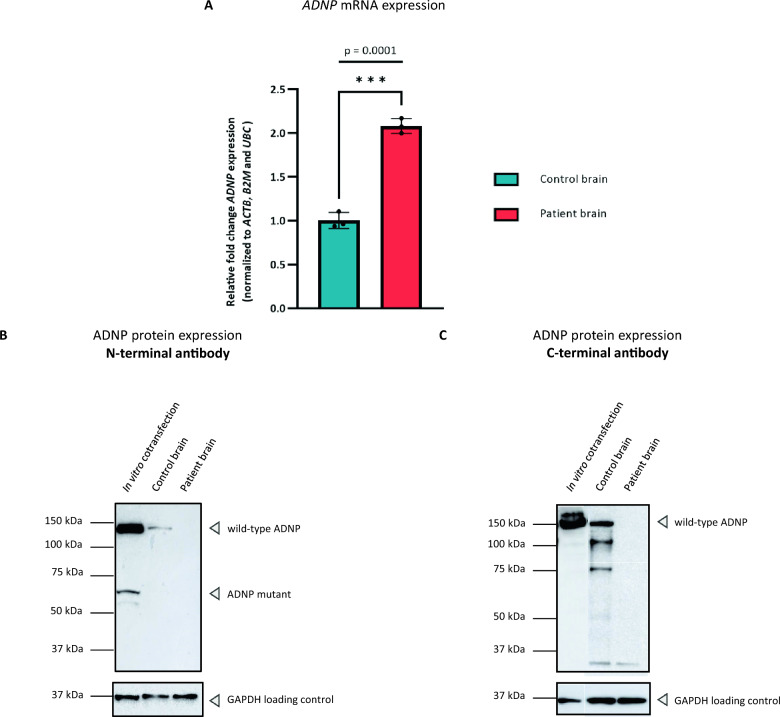
ADNP expression analysis of the cerebellum. (**A**) RT-PCR showing a significant increase in *ADNP* mRNA levels in the patient cerebellum compared to an age-and sex matched control subject (***p = 0.0001; unpaired student T-test). Gene expression values were normalized with three stable reference genes, i.e., *β-Actin* (*BACT*), *β-2-Microglobulin* (*B2M*), and *Ubiquitin C* (*UBC*). **(B)** ADNP protein expression analysis using an N-terminal antibody. Western blotting showed the presence of wild-type ADNP (150 kDa) in overexpression lysates with presence of the truncated protein. However, expression was absent in the patient as compared to the control, where wild-type ADNP could be visualized. **(C)** ADNP protein expression analysis using a C-terminal antibody. Western blotting showed the presence of wild-type ADNP (150 kDa) in overexpression lysates, as well as in the control subject, but not in the patient. GAPDH was used as a loading control for normalization

Cerebellar tissue, known for its highest *ADNP* expression [35], allowed to validate the presence of *ADNP* mRNA and protein in autopsy material by performing an expression analysis using real time reverse-transcription PCR (RT-PCR) and Western blotting. To investigate wild-type *ADNP* mRNA levels, we designed a primer set at the 3’ region of exon 6 (corresponding to the C-terminal portion of the protein). Here, a significant two-fold increase in the total *ADNP* levels was observed in the patient compared to the control subject (*p* = 0.0001; ***), consistent with findings in our RNA sequencing described below (Fig. 2A). Attempts to quantify the 5’ end of the transcript were not successful, suggesting partial mRNA degradation. At the protein level, we tested endogenous ADNP levels in the human brain using extensively validated C-terminal and N-terminal ADNP antibodies [29]. We were able to detect wild-type ADNP levels (150 kDa) in the control brain, but not in the patient using both antibodies (Fig. 2B, C). To investigate the co-expression of the full length and mutant protein, we co-transfected wild-type and p.His559Glnfs*3 mutant N-DYKDDDDK (Flag®) expression vectors in HEK293T cells. Co-expression of wild-type and mutant ADNP demonstrated the presence of the wild-type protein (150 kDa) together with a truncated mutant protein (63 kDa) using an N-terminal antibody, mimicking the expected expression in the patient. C-terminal antibody incubation resulted in the detection of the wild-type ADNP (150 kDa) exclusively. Together, these findings confirm a molecular weight of ADNP (150 kDa), above its calculated molecular weight of 123 kDa, but show instability of the protein in post-mortem brain material of the patient.

**Fig. 3:**
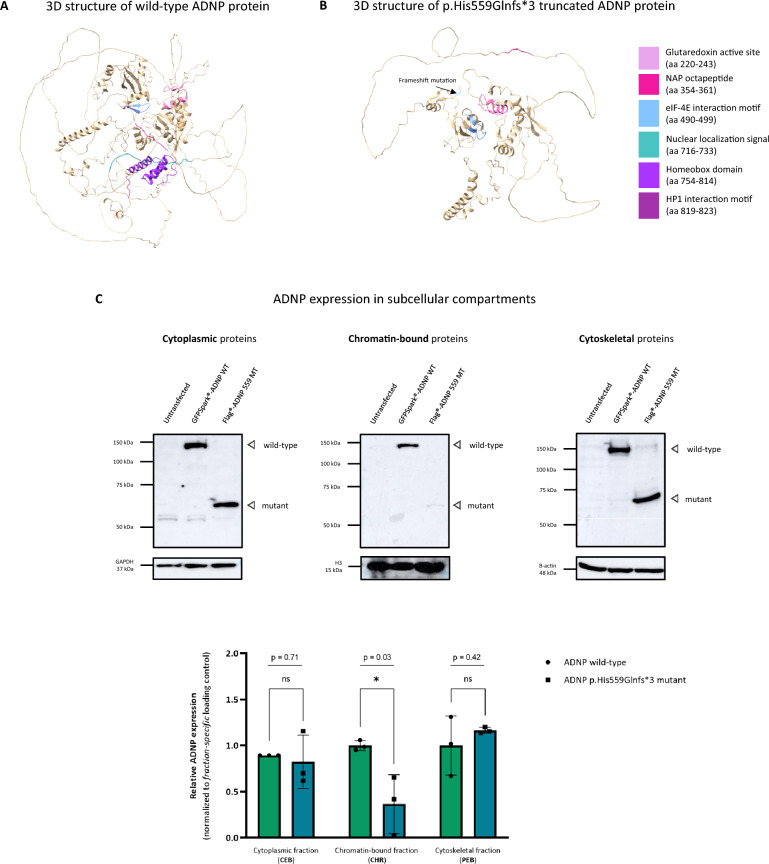
The ADNP patient mutation impairs expression in the chromatin-enriched protein fraction. (**A**) 3D protein structure representation of the wild-type ADNP glutaredoxin active site (pink), NAP octapeptide sequence (fuchsia), eIF-4E interaction motif (blue), nuclear localization signal (dark cyan), homeobox domain (blue violet), and HP1 interaction motif (purple). The NAP domain (fuchsia) presents at the surface of the protein. (**B**) The nuclear localization signal-truncating p.His559Glnfs*3 mutant shows loss of the HP1-binding motif and DNA homeobox domain. (**C**) N-terminal ADNP detection in different subcellular compartments normalized to their protein fraction-specific loading controls. Detection of wild-type N-DYKDDDDK (Flag®)-tagged ADNP shows a molecular weight of 150 kDa. The p.His559Glnfs*3 mutant showed a lower molecular weight of 63 kDa. Cytoplasmic enrichment shows expression of wild-type ADNP (150 kDa) and the mislocalized p.His559Glnfs*3 mutant (63 kDa) with no difference in expression (*p* = 0.71; ns). Chromatin-enriched fraction demonstrated partial loss of mutant ADNP levels compared to wild-type ADNP, showing a dramatic decrease in expression (*p* = 0.03; *). Cytoskeletal fraction is enriched for wild-type ADNP and the p.His559Glnfs*3 mutant, with no significant difference in expression (*p* = 0.42; ns). GAPDH (cytoplasmic fraction), histone H3 (chromatin-bound fraction), and β-actin (cytoskeletal fraction) were used as loading controls. Statistical analysis of the subcellular fractionation immunoblots was performed using an unpaired two-tailed student T-test, assuming equal variances

To study the molecular impact of the patient mutation, we performed in silico modeling of the wild-type ADNP protein (UniProt; Q9H2P0) and p.His559Glnfs*3 mutant using AlfaFold. Here, the structure of the wild-type protein demonstrated the DNA-binding homeobox domain in proximity to the bipartite NLS sequence, whereas the neuroprotective NAP motif resides at the surface of the protein, being partially occluded by flexible intrinsically disordered regions (IDRs) and low-complexity regions (LCRs) located near the C-terminus, suggestive for a role for protein–protein interactions [36, 37]. Moreover, the eIF-4E binding motifs and the glutaredoxin active site are centrally positioned in the core of the wild-type protein, assembling several of its zinc finger motifs (Fig. 3A). The p.His559Glnfs*3 mutant truncates the NLS region, impairing nuclear transport [11]. Moreover, downstream protein domains, including the DNA-binding homeodomain and the HP1 binding motif are also lost as a result of the truncating mutation. Overall, the p.His559Gln*3 mutant lacks some of the IDRs but has a similar structural confirmation compared to the wild-type protein (Fig. 3B). Subsequently, we examined stable ADNP protein levels in several subcellular compartments including the cytoplasm, nucleus with chromatin-enriched proteins, and the cytoskeleton in HEK293T overexpression lysates. In the cytoplasm, we detected wild-type (150 kDa) and mutant (63 kDa) ADNP using an N-terminal antibody showing no significant difference in expression levels (*p* = 0.71; ns). In the chromatin-bound fraction, we visualized the wild-type and mutant protein with a significant decrease of mutant protein levels (*p* = 0.03; *). Moreover, we demonstrated the expression of mutant and wild-type ADNP in the cytoskeletal protein fraction. However, we did not observe a significant difference (*p* = 0.42; ns) in the expression of the mutant compared to the wild-type protein (Fig. 3C).

### Genome-wide methylation analysis of the cerebellum demonstrates abnormalities of the cytoskeleton and autophagy together with an aberrant transcription factor function of ADNP during development

**Fig. 4:**
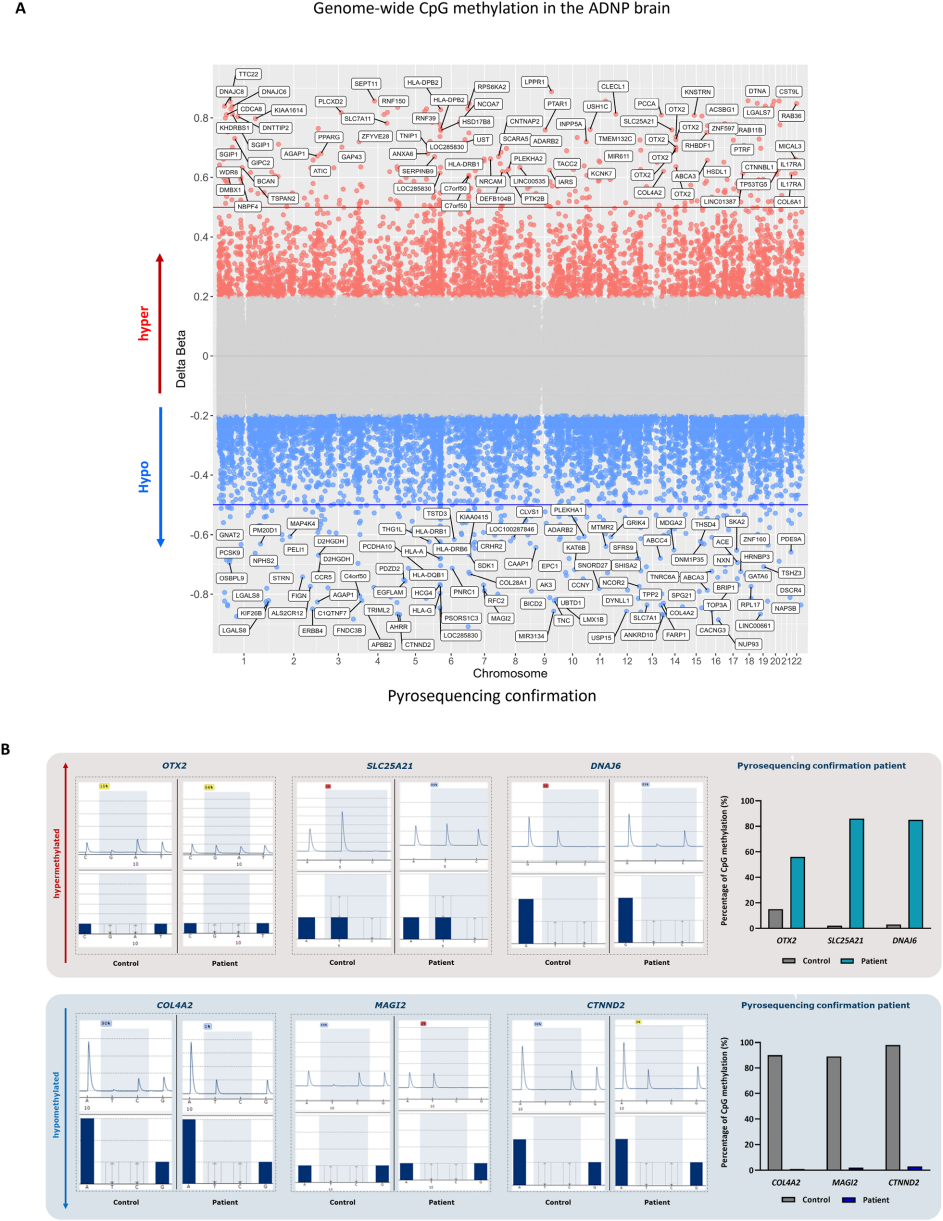

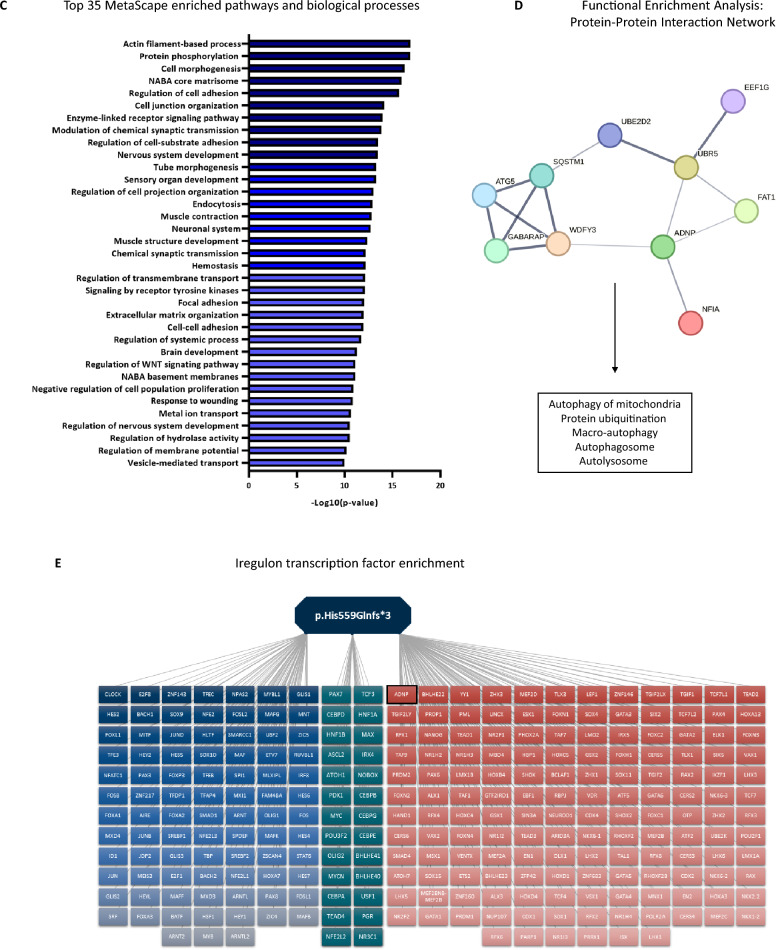
ADNP methylation signature in the juvenile post-mortem cerebellum. (**A**) Genomic scatter plot indicating the hypermethylated genes (Δβ > 0.2) of the patient (red), the hypomethylated genes (Δβ < −0.2) of the patient (blue). The chromosomal positions of the genes are shown on the x-axis. (**B**) Pyrosequencing confirmation of a subset of hyper- and hypomethylated genes. Hypermethylated genes, e.g., *OTX2*, *SLC25A21* and *DNAJ6*, show increased CpG methylation in the patient, whereas hypomethylated genes, e.g., *COL4A2*, *MAGI2* and *CTNND2*, present with a nearly absent percentage of CpG methylation. (**C**) Metascape functional annotation of biological processes. Hyper- and hypomethylated genes cluster in associated processes such as the actin cytoskeleton and nervous system developmental disorder amongst others. (**D**) Predictive String v11.5 protein–protein interaction analysis of ADNP. The proteins are indicated as nodes with interconnecting lines representing the interaction. ADNP is surrounded by protein regulating specific autophagy-related processes and protein ubiquitination. (**E**) Transcription factors (TFs) enriched in patient cerebellum of hyper- and hypomethylated gene co-expression. TFs associated with hypermethylated genes are represented in blue, while the TFs associated with the hypomethylated genes are depicted in red. TFs shared amongst the overlapping genes are shown in green. *ADNP* was identified as the top transcription factor controlling the hypomethylated genes (black box)

As methylation signatures are robust and even conserved in ancient DNA [38], we decided to start our exploration by performing an EPIC BeadChip array on the cerebellum of the died ADNP patient and an age-matched control brain. Here, we show enrichment of 6289 CpG probes with a minimum 20% difference in methylation in the ADNP patient compared to the control. Specifically, we identified 2394 CpG probes showing hypermethylation (Δβ > 0.2), whereas a vast amount of 3895 CpG probes were hypomethylated (Δβ < −0.2). In addition, 1547 hypermethylated gene probes could be annotated to 1162 genes, while 2500 hypomethylated gene probes were associated with 1842 genes (Additional file 3: Data S1), indicating a Class I episignature [18, 19], extending findings from peripheral blood to the human brain for the first time (Fig. 4A). Next, we confirmed a selection on genes prioritized for methylation in the 5’UTR, 3’ UTR and transcription start site (TSS) together with associations to autism or other Helsmoortel–Van der Aa syndrome-related clinical features. We selected the hypermethylated genes *OTX2, SLC25A21,* and *DNAJ6* and the hypomethylated genes *COL4A2, MAGI2,* and *CTNND2* for pyrosequencing. Here, we could confirm a higher percentage of CpG methylation in the patient for *OTX2 (56%), SLC25A21 (86%),* and *DNAJ6 (85%)* compared to the control subject. Respectively, we could also demonstrate a lower percentage of CpG methylation in the patient for *COL4A2 (1%), MAGI2 (2%),* and *CTNND2 (3%)* (Fig. 4B). Next, we performed functional annotation of the hyper- and hypomethylated genes using Metascape. Enriched biological processes and GO terms included actin filament-based processes, cell adhesion, nervous system development, muscle contraction, brain development, the WNT signaling pathway, regulation of membrane potential, and synaptic transmission amongst others (Fig. 4C). Functional enrichment analysis for protein–protein interactions was predicted for ADNP using the STRING database. We identified four suggested interactions of ADNP with WDFY3, UBR5, FAT1, and NFIA, which play a role in autophagy of the mitochondria, protein ubiquitination, macro-autophagy, autophagosome and autolysosome formation (Fig. 4D). Given the role of *Adnp* as a putative transcription factor [35, 39], we performed a transcription factor enrichment of both hyper- and hypomethylated genes. Here, we identified a module of 44 co-expressed genes, which were subsequently inserted in CytoScape using the IRegulon function for TF enrichment (Additional file 4: Data S2). We observe a stronger enrichment of TFs associated with hypomethylated genes (red) than hypermethylated genes (blue) and shared TFs (green). Among the upregulated TFs associated with hypomethylated genes presented pluripotency and cell fate-determining genes such as *POU2F1*, *TEAD2*, *SOX1/4*, *GATA1/2/3/5/6, PAX4/6, NANOG, and NEUROD1*, as well as chromatin modifiers like *YY1, SIN3A* and *ADNP* itself. On the other hand, the downregulated TF cluster associated with hypermethylated genes was also enriched for *PAX and SOX*-related genes, indicating abnormal lineage specification of neuronal progenitor cells. The shared TF cluster showed presence of *HNF1A*, a gene controlling expression of several liver-specific genes (Fig. 4E). Our genome-wide cerebellar methylation analysis indicates strong molecular evidence for a deregulated function of *ADNP* as a transcription factor, impacting lineage specification and genes implicated in brain development.

### RNA sequencing substantiates downregulation of the WNT signaling pathway and autophagy defects in cerebellar autopsy tissue

**Fig. 5:**
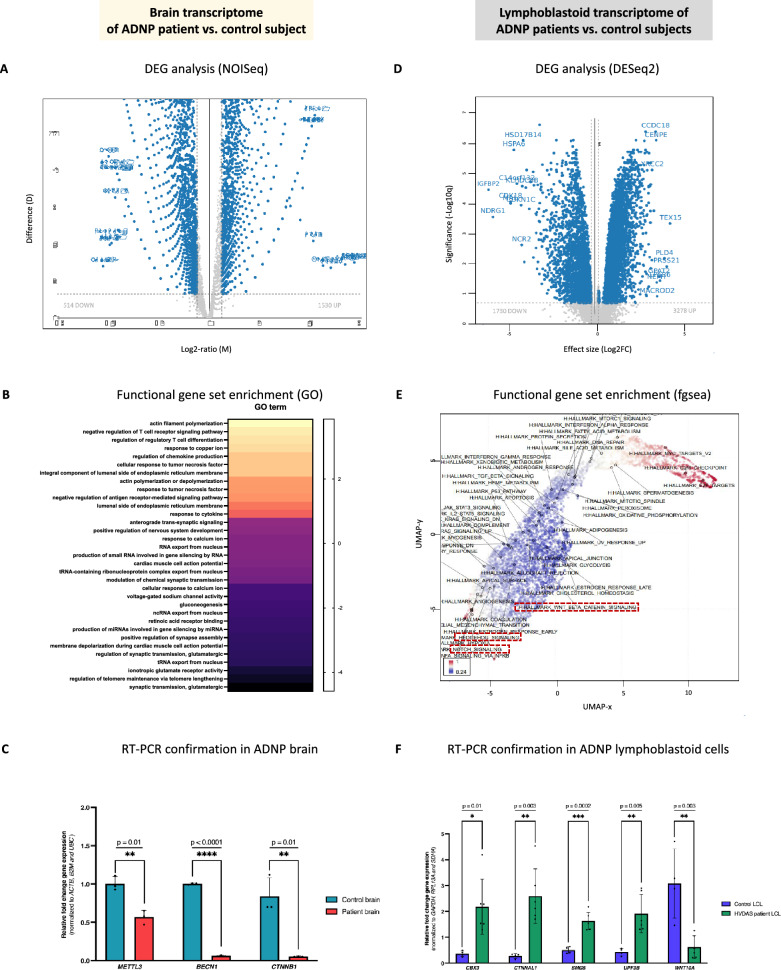
Cerebellar and lymphoid gene expression changes are associated with different *ADNP* mutations. (**A**) Volcano plot of differentially expressed genes (DEGs) in the ADNP cerebellum using the NOISeq algorithm, representing the effect size M (log2 ratio) and D (difference between conditions) values. The DEG are shown in blue. (**B**) Gene set enrichment analysis of all DEGs in gene ontology (GO), biological processes (BP) and molecular function (MF) reveals specific Helsmoortel–Van der Aa syndrome-related pathways in the ADNP brain. (**C**) RT-PCR showing a significant reduction of *METTL3*, *BECN1* and *CTNNB1* mRNA levels in the ADNP cerebellum compared to the age-matched control subject. Expression values were normalized using the housekeeping genes *ACTB*, *B2M* and *UBC*. Data was analyzed using an unpaired student T-test. (**D**) Volcano plot of DEGs in the patient LCLs using the DESeq2 package, displaying the significance (-log10q) and effect size (log2FC). The DEG are shown in blue. (**E**) Functional gene set enrichment of GO and BP using differentially expressed genes in the ADNP LCLs as compared to age- and sex-matched controls. UMAP clustering of gene sets colored by standard deviation, variance, or mean fold-change in patient LCLs shows clear downregulation of the WNT, Hedgehog and Notch signaling pathways (marked in a red box), impairing proper neuronal development. Downregulated genes, blue; upregulated genes, red. (**F**) RT-PCR showing a significant increase of *CBX3*, *CTNNAL1, SMG5* and *UPF3B* together with a significant decrease in *WTN10A* mRNA levels in patients versus control LCLs. Expression values were normalized using the housekeeping genes *GAPDH*, *RPL13A* and *SDHA*. Data was subsequently analyzed with a Mann–Whitney U test for unpaired measures

To determine differential expression beyond methylation differences, we performed bulk transcriptome sequencing of cerebellar tissue of the ADNP autopsy. As RNA is much less stable over time, we first performed an extensive quality control by evaluating total RNA purity and integrity (see experimental methods). Using bulk mRNA sequencing, we determined the gene ratio (patient/control) using the NOISeq algorithm, a non-parametric method for comparing samples without biological replicates, reporting the log2-ratio of the two conditions (M) and the value of the difference between the conditions (D) [40]. We tested for differential expression across all 7659 genes that appeared in our data set (Additional file 5: Data S3). In line with the observation of an excess of hypomethylated CpG probes, we observed an excess of upregulated genes. Using a significance cut-off equivalent to, *p* value < 0.05, FDR =  < 0.05, and a biologically meaningful (M-value) log2FC > 0.5, we found 514 downregulated and 1520 upregulated genes with differential expression (Fig. 5A). Gene expression alterations in the ADNP cerebellum were notable with the majority of genes presenting with an M-value < 5. Gene ontology (GO) enrichment revealed downregulation of glutamatergic synaptic transmission, abnormal cardiac muscle cell conductivity, and nervous system development, whereas cytoskeleton dynamics were upregulated. A remarked enrichment of immune system-related responses was observed that are potentially related to the patient’s immunosuppressant treatment (Fig. 5B). We confirmed a selected set of genes with RT-PCR, including the RNA-methylation gene *METTL3* (*p* = 0.005; **), autophagy inducer *BECN1* (*p* < 0.0001; ****), and WNT signaling ligand *CTNNB1* (*p* = 0.001; **) (Fig. 5C). To better interpret the differential expression in the ADNP brain, we compared the transcriptome analysis of the autopsy with the differential expression observed in immortalized LCLs of multiple patients with different *ADNP* mutations. We tested for differential expression across approximately 10,000 protein-coding transcripts that appeared in our data set (Additional file 6: Data S4). Using the exact cut-off criteria as in the autopsy, we found 1730 downregulated and 3278 upregulated genes with differential expression, indicating that the *ADNP* mutations rather induce gene upregulation (Fig. 5D). Fast Gene Set Enrichment Analysis (fgsea) identified similar molecular pathways as identified in the autopsy (Fig. 5E). We confirmed a subset of five genes with RT-PCR, including the heterochromatin marker and ADNP-interacting gene *CBX3* (*p* = 0.01; *), WNT signaling member *WNT10A* (*p* = 0.003; **), actin-cadherin mediator *CTNNAL1* (*p* = 0.003; **) as well as nonsense mediated decay members *SMG5* (p = 0.0002; ***) and *UPF3B* (*p* = 0.005; **) (Fig. 5F).

**Fig. 6:**
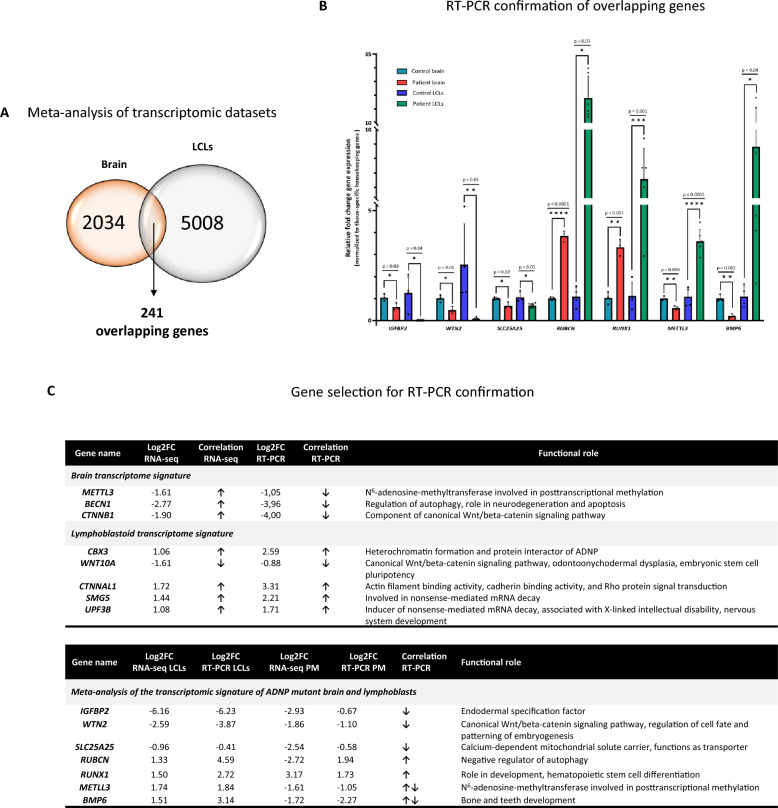
Meta-analysis of the transcriptomic signature identified in the *ADNP* brain and lymphoblasts. (**A**) Venn diagram representing the exact amount of DEGs in the human ADNP cerebellum and lymphoblastoid data sets, converging to an amount of 241 overlapping genes. (**B**) RT-PCR showing a significant decrease of *IGFBP2*, *WNT2, SLC25A25* together with a significant increase in *RUBCN* and *RUNX1* mRNA levels in patient brain and patient LCLs as compared to their age-matched controls. Note a difference in mRNA expression of *BMP6* and *METTL3* amongst brain tissue and LCLs. Expression values were normalized using the tissue-specific housekeeping genes (mentioned above). Data was subsequently analyzed with an unpaired student T-test (brain) or Mann–Whitney U test for unpaired measures (LCLs). (**C**) Correlation of DEGs from the NOIseq and DESeq2 analysis (RNA-seq) and RT-PCR confirmations with their functional cellular role. Selected genes are represented with their Log2FC and compared for overlap with RT-PCR. Arrow upwards, upregulation; arrow downwards, downregulation

To investigate the potential impact of the *ADNP* mutation in the human brain, we intersected the DEGs identified in both data sets (Additional file 7: Data S5), which revealed an overlap of 241 genes between the ADNP autopsy brain and LCLs (Fig. 6A). We observed a striking resemblance for biological relevance of genes involved in endoderm specification *IGFBP2* (brain, *p = 0.03; LCL, *p = 0.04), canonical WNT signaling *WNT2* (brain, *p = 0.01; LCL, **p = 0.01), mitochondrial transporter *SLC25A25* (brain, *p = 0.02; LCL, *p = 0.03), autophagy regulation *RUBCN* (brain, ****p < 0.0001; LCL, *p = 0.003), hematopoietic stem cell differentiation *RUNX1* (brain, **p = 0.001; LCL, ***p = 0.001), N^6^-adenosine-methylation *METTL3* (brain, **p = 0.005; LCL, ****p < 0.0001), and bone and teeth development *BMP6* (brain, **p = 0.002; LCL, *p = 0.04) (Fig. 6B, C). In conclusion, these robust gene expression changes related to the nervous system and morphogenesis underline a regulating role of ADNP in the human brain and blood of patients, confirmed by salient pathways including the WNT signaling, autophagy, and bone development together with involvement in processes such as hematopoietic stem cell differentiation and unexpected RNA methylation.

### Shotgun proteomics links chromatin remodeling to autophagy in the ADNP autopsy brain

**Fig. 7:**
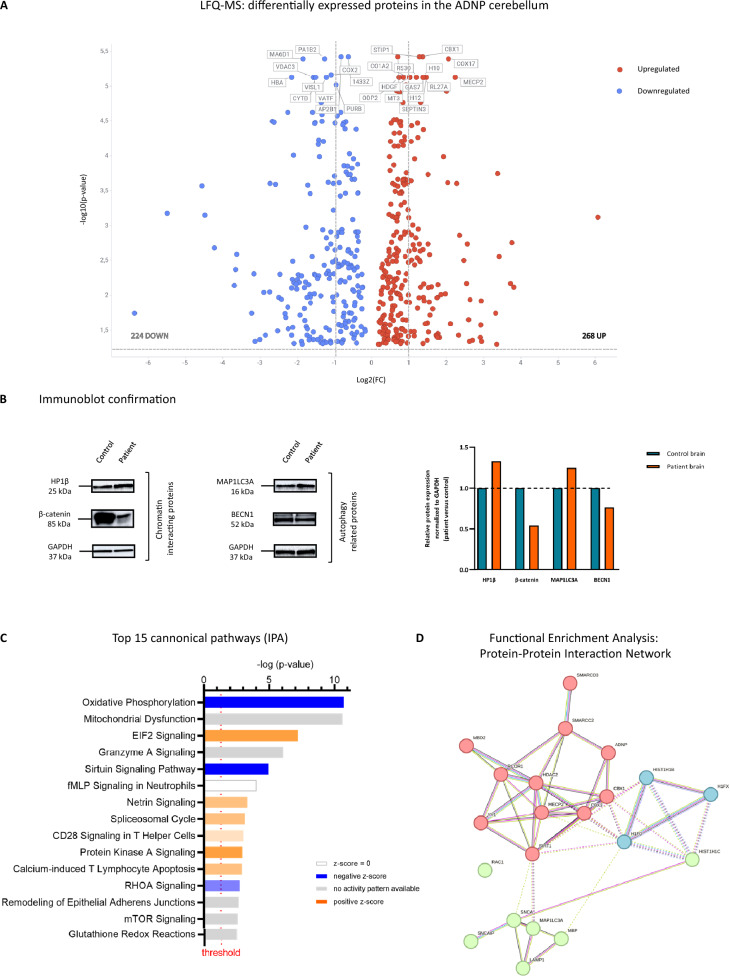
Discovery of SIRT1 interactions in the autistic brain, linking chromatin remodelers to autophagy. (**A**) Volcano plot of differentially expressed proteins (DEPs) in the ADNP patient cerebellum, represented by the significance (−log10(*p*)) and effect size (log2FC). Proteins with a significant downregulation are shown in blue and those with an upregulation in red. Note the marked difference in expression of the ADNP-interacting protein CBX1/HP1β. (**B**) Immunoblot confirmations of DEPs exposed to their specific antibodies. (**C**) Top 15-ranked canonical IPA pathways of the ADNP brain, represented as z-scored expression values and significance (−log *p*-value). Activated pathways are presented in orange, respectively lowered activity in blue; no activity pattern available (grey); pathways with no difference in activity (white). (**D**) A predictive protein–protein interaction network was generated by String Version 11.5, integrating associations of a co-expression hub identified amongst the DEPs. Proteins are represented by colored network nodes in relation to each other with SIRT1 fulfilling a centralizing role. The edges illustrate functional associations and the lines between the nodes represent the existence of evidence for associations

As post-transcriptional regulation can further increase variation in gene expression levels [41], proteome analysis was performed by label-free quantitation (LFQ) mass spectrometry on the cerebellum to study the effect of the c.1676duplA/p.His559Glnfs*3 *ADNP* mutation at protein expression level. Chromatographic conditions between different runs were highly reproducible, resulting in a strong correlation between LFQ intensities and technical replicates (Additional file 8: Data S6). Overall, we detected approximately 1522 protein groups per sample under a 1% false-discovery rate (FDR) with fixed modifications of carbamidomethylation (C), deamidation (QN) and oxidation (M). Moreover, we identified 4552 proteins with more than two unique peptides, respectively 988 proteins with at least two unique peptides, and 1477 with one unique peptide. Next, we used MetaboAnalyst 5.0 to quantify differences detected in patient versus control cerebellum. Among the 2455 quality-filtered proteins, we detected 492 proteins with a differential expression (Additional file 9: Data S7), of which 224 proteins were significantly downregulated, while 268 proteins showed a significant upregulation in the post-mortem patient cerebellum (two-tailed student T-test; padj < 0.05). Next, we plotted the top 10 downregulated (represented in blue) and upregulated (represented in red) proteins identified in patient versus control brain, showing a clear upregulation of the major ADNP-interacting protein heterochromatin Protein 1 homolog beta (CBX1/HP1β), amongst others, indicating that ADNP is able to somehow affect the expression of its direct interaction partner (Fig. 7A). Subsequently, we applied immunoblot experiments to confirm the downregulation of β-catenin and BECN1 protein levels in the patient brain, in line with its decreased transcription levels. Surprisingly, we also observed differential expression of an additional autophagy marker, MAP1LC3A, in the ADNP brain consistent with aberrant autophagy defects in our transcriptome data (Fig. 7B). Clustering of the differentially expressed proteins (DEPs) in canonical pathways using IPA indicated a decreased activity of mitochondrial oxidative phosphorylation, sirtuin signaling and RhoA signaling. In contrast, IPA predicted an increase in EIF2 signaling, spliceosomal cycle and protein kinase A signaling in the patient. We also observed an enrichment of pathways with no difference in activity, including granzyme A signaling and mTOR signaling, T-helper signaling, and apoptosis (Fig. 7C). Next, we mapped all DEPs in a functional enrichment analysis and predicted possible protein–protein interactions of ADNP with the identified DEPs as well as with other biologically correlated proteins. Of particular interest, the histone deacetylase sirtuin 1 (SIRT1) in the center of the protein network was found to link various chromatin modifier proteins such as MECP2, ADNP, SMARCC2, HDAC2 including YY1, and chromatin-associating proteins such as CBX1/3, histones H1F0 and H1.2 to autophagy regulators like MAP1LC3A and LAMP1 (Fig. 7D). In this section, we showed that the proteomic landscape of ADNP brain autopsy material corroborates our transcriptome findings, e.g., upregulation of ADNP-interactor CBX1/HP1β together with a downregulation of β-catenin and BECN1, supported by abnormalities attributed to the WNT signaling pathway and autophagy.

### ADNP and SIRT1 co-immunoprecipitate with the microtubule end-binding proteins 1 and 3 (EB1/EB3)

**Fig. 8:**
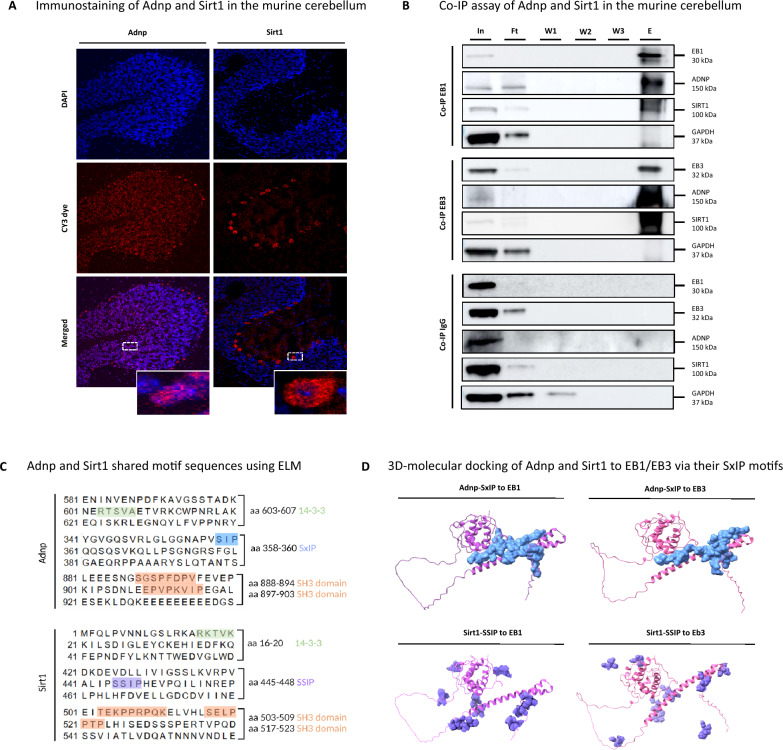
ADNP indirectly interacts with the histone deacetylase enzyme SIRT1 through the microtubule end-binding proteins EB1/EB3. (**A**) Adnp and Sirt1 immunostaining (red Cy3 fluorescence) in cryosections of the murine cerebellum was assessed by confocal scanning microscopy. Adnp was mainly observed in the nucleus and Sirt1 was mostly located in the cytoplasm. (**B**) Co-IP assay of Adnp and Sirt1 in the murine cerebellum. IP-competent EB1 and EB3 antibodies were crosslinked to agarose beads and sequentially eluted in fractions (input, In; flow-through, Ft; three consecutive washes, w1-w3; and elution, E). All fractions were analyzed by western blotting for Adnp, Sirt1, EB1, and EB3. IgG non-reactive beads were used as a negative control. GAPDH has been used as loading control for all western blots, and critical assessment of the accuracy of the Co-IP method. (**C**) ELM analysis identified shared motif sequences of Adnp and Sirt, including 14–3–3 motifs (green), SxIP motif (blue), SH3 domains (orange), and the SSIP motif (violet). (**D**) In silico 3D-molecular docking of Adnp (SxIP motif) to EB1/3, left (purple) and right (pink) respectively to Sirt1 in the panel below (SSIP motif) to EB1/3, left (purple) and right (pink)

Recently, various studies identified an association between mitochondrial dysfunction, autophagy regulation, and autism spectrum disorders [42–44]. Similarly, our proteomic protein–protein interaction study mapped SIRT1 at the crossroads of chromatin remodelers and autophagic regulators in the ADNP autopsy brain. Besides, SIRT has been discovered to maintain genomic stability [45], to enhance synaptic plasticity [46], to suppress inflammation [47], to fulfill a neuroprotective function [48], and to positively regulate autophagy and mitochondrial function [49]. In addition, SIRT1 is known to modulate chromatin structure by activating BRG1, which is a chromatin remodeling interaction partner of ADNP in the SWI/SNF complex [9, 50]. Hence, we reasoned that ADNP and SIRT1 may share common regulatory partners in chromatin remodeling and microtube dynamics that regulate autophagy. To further validate a direct protein interaction of ADNP and SIRT1 in the human brain, co-immunoprecipitation (co-IP) experiments were performed. However, due to the instability of the ADNP protein, co-IP experiments were not successful. Therefore, we alternatively demonstrated the subcellular localization of Adnp and Sirt1 by immunostaining the cerebellum dissected from male C57BL/6JCr wild-type mice as a model for the human condition. Here, cerebellar cryosections were immunostained with primary monoclonal ADNP and SIRT1 antibodies (Cy3 red fluorescent signal), and nuclei were counterstained with DAPI (blue). Adnp expression was predominantly detected in the nucleus, with occasional weak cytoplasmic signals, visualized by the overlap of the red Adnp signal and blue DAPI counterstaining. In contrast, Sirt1 was predominantly situated in the cytoplasm of Purkinje cells in the cerebellum, with occasional nuclear immunoreactivity (Fig. 8A). Indeed, an indirect interaction of ADNP and SIRT1 was shown in SH-SY5Y cells which could not be validated in human induced pluripotent stem cells (hIPSC)-derived neuronal differentiated cells [16]. Therefore, we next investigated the potential indirect interaction of Adnp and Sirt1 through the EB1/EB3 proteins in murine cerebellar brain lysates with a co-immunoprecipitation assay. During this process, we performed stringent washing steps using high detergent buffers to prevent false-positive binding. In addition, we also controlled each western blot with GAPDH, whose intensity was absent after co-immunoprecipitation of the bait protein. We observed specific co-immunoprecipitation of Adnp (150 kDa) and Sirt1 (100 kDa) in the presence of both EB1 (30 kDa) and EB3 (32 kDa) antibodies. IgG non-reactive beads were used as a negative control, showing no immunoreactivity of Adnp, Sirt1 together with EB1 and EB3 in the eluted fraction (Fig. 8B). To better understand the physical connections between Adnp (UniProt; Q9Z103) and Sirt1 (UniProt; Q53Z05), we applied a eukaryotic linear motif (ELM) analysis to unravel shared motifs (Fig. 8C). Interestingly, as partially shown before, we identified a series of common interaction motifs, including (1) SxIP motif for Adnp (aa 354–360, NAPVSIP, *p* = 0.01) and similar SSIP for Sirt1 (aa 440–448, VALIPSSIP, *p* = 0.0002), (2) SH3-domains for Adnp (i.e., aa189–195, FQHVAAP, *p* = 0.01) and Sirt1 (i.e., aa 506–511, PPRPQK, *p* = 0.001), and (3) 14–3–3 motifs for Adnp (i.e., aa 16–20, RKTVK, *p* = 0.004) and Sirt1 (i.e., aa 333–342, RNYTQNIDTL, *p* = 0.004). The presence of a SxIP motif is a unique feature ascribed to both ADNP and SIRT1, as only 42 protein have been identified by mass-spectrometry based methods to contain this conserved motif [51, 52]. Original studies have identified the microtubule-end binding proteins EB1 and EB3 as interaction partners of ADNP through its SxIP motif [16, 23, 27]. Along the same line, we predicted a physical interaction between Adnp, Sirt1 and EB1/EB3 in silico via 3D-molecular docking. Upon ranking the models according to the amount of interacting amino acids, the top 10 docking interactions were derived from ClusPro and processed in ChimeraX. Molecular docking revealed possible Adnp (blue) binding to both microtubule-end binding proteins EB1 (left, violet) and EB3 (right, pink) via amino acids 358–360, corresponding to its SxIP motif. In addition, Sirt1 was predicted to interact with EB1 (left, violet) and EB3 (right, pink) proteins through its similar SSIP motif at amino acid position 446–448 (Fig. 8D). In conclusion, our findings suggest that Adnp and Sirt1 might indirectly co-immunoprecipitate in the presence of the EB1/EB3 proteins via the SxIP motif for ADNP, respectively SSIP for SIRT1.

### The ADNP-EB1/EB3-SIRT1 complex regulates mitochondrial autophagy (mitophagy) and respiratory functions

**Fig. 9:**
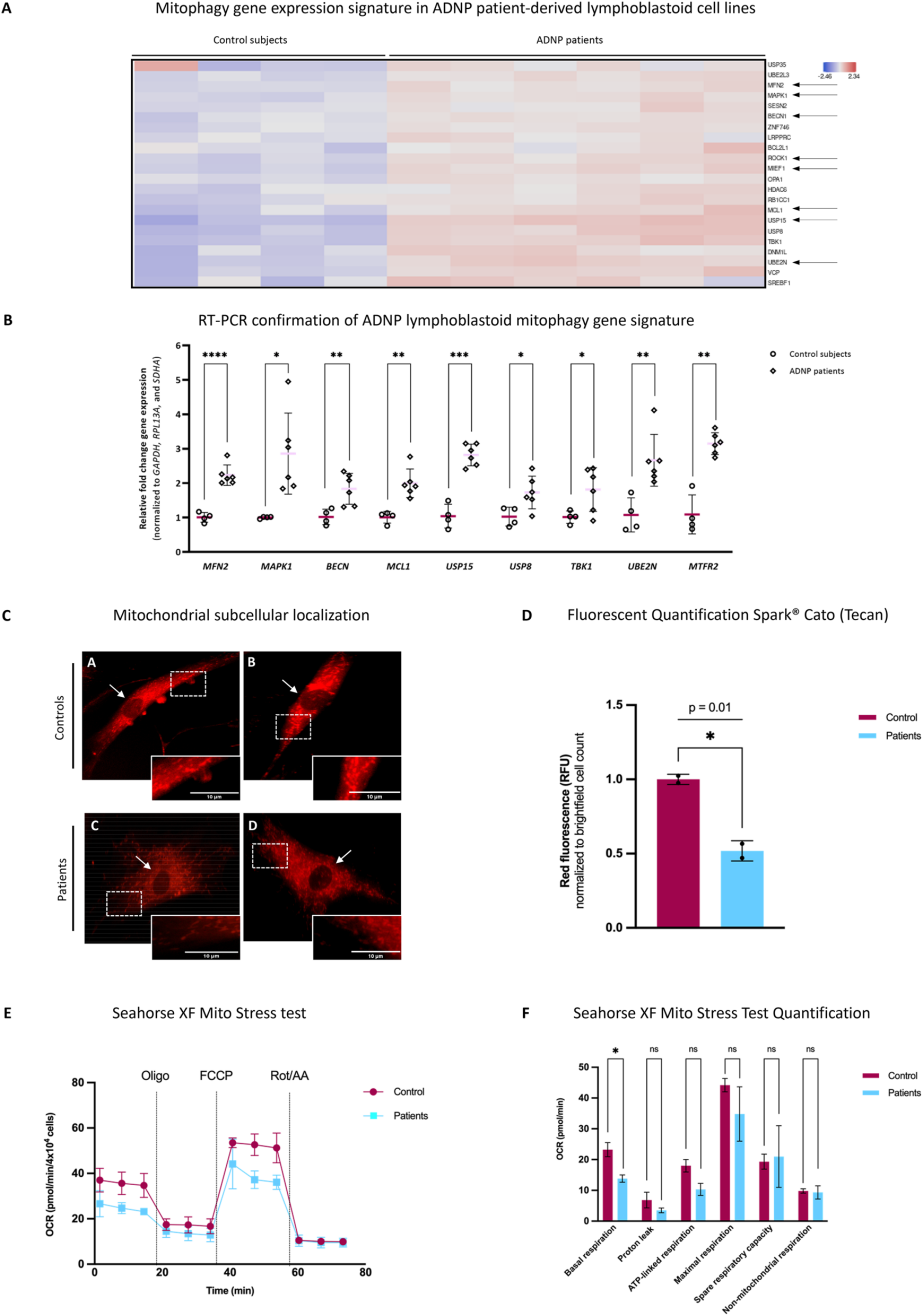
Mitophagy gene signature and mitochondrial impairments in *ADNP* patient-derived cell lines. (**A**) Expression levels of mitophagy-related genes in *ADNP* patient LCLs compared to age and sex-matched controls. mRNA sequencing demonstrated an upregulated mitophagy gene signature in patient LCLs as compared to controls. (**B**) RT-PCR showing a significant increase of mitophagy-related genes in ADNP patients versus control LCLs. Expression values were normalized using the housekeeping genes *GAPDH, RPL13A* and *SDHA*. Data was subsequently analyzed with an unpaired student T-test assuming unequal variances with a Welch’s multiple testing correction. (**C**) Subcellular mitochondrial distribution using the MitoTracker® Red CM-H_2_XRos fluorescent staining of control subjects (A-B) and *ADNP* patient (C-D) fibroblasts. The white arrow indicated the unstained nucleus of the fibroblast. (**D**) MitoTracker® red fluorescent signal (RFU) was normalized to brightfield cell count and quantified in patient and control fibroblasts using a multimode microplate reader (Tecan Spark™) at an excitation of 485 nm. A significant decrease (*p* = 0.01, student T-test) in red fluorescent signal, corresponding to the mitochondrial activity, was observed in patient-derived fibroblasts (blue) compared to the control cells (red). (**E**) The Seahorse Cell Mito Stress Assay was used to measure changes in oxygen consumption rate (OCR) in patient-derived (blue) and control (red) fibroblasts after different triggers that inhibit or activate mitochondrial respiration. Oligo = oligomycin, a complex V inhibitor to decrease the electron flow through the electron transport chain (ETC); FCCP = Carbonyl cyanide-*p*-trifluoromethoxyphenylhydrazone, the uncoupling agent to promote maximum electro flow through of the ETC; Rot/AA = rotenone and antimycin, complex I and complex II inhibitors respectively, to shut down the mitochondrial-related respiration. (**F**) Based on the changes in OCR, several aspects of the mitochondrial respiration could be quantified using the Agilent Seahorse analytics software. Statistical significance was calculated using an unpaired student T-test assuming equal variances. Data is shown as mean ± sd. The basal respiration (*p* = 0.04, *), proton leak (*p* = 0.21; ns), ATP-linked respiration (*p* = 0.06, ns), maximal respiration (*p* = 0.28, ns), spare capacity (*p* = 0.84, ns), and non-mitochondrial respiration (*p* = 0.79; ns) were indicated for patients (blue) compared controls (red)

RNA sequencing data of the ADNP brain autopsy was subsequently analyzed for the enrichment of specialized pathways associated with autophagy [53, 54], neuroprotection [55, 56], and mitochondrial biogenesis [57] with a costomized gene toolbox [34]. By this approach, we found enrichment of genes involved in mitophagy (Additional file 1: Table S5), an autophagy-dependent process regulating mitochondrial homeostasis [42]. Similarly, RNA sequencing data of the ADNP patient LCLs also revealed enrichment of mitophagy-related genes e.g., *ubiquitin Specific Peptidase 15* (*USP15*), *mitochondrial distribution and morphology regulator 1* (*MSTO1*), *mitochondrial fission regulator 2* (*MTFR2*), *apoptosis inducing factor mitochondria associated 1* (*AIFM1*), *Mitofusin 2* (*MFN2*), *Beclin 1* (*BECN1*), and *mitochondrial Elongation Factor 1* (*MIEF1*) (Fig. 9A). RNAseq results of nine DEGs were further validated by RT-PCR, including *MFN2* (*p* < 0.0001; ****), *MAPK1* (*p* = 0.01; *), *BECN1* (*p* < 0.006; **), *MCL1* (*p* = 0.001; **), *USP15* (*p* = 0.0002; ***), *USP8* (*p* = 0.02; *), *TBK1* (*p* = 0.03; *), *UBE2N* (*p* = 0.004; **), and *MTFR2* (*p* = 0.002; **) (Fig. 9B). Since we obtained two reciprocally regulated mitophagy gene clusters in the human brain (Additional file 10: Data S8), we next determined the autophagic flux [58, 59] by Bafilomycin A1 treatment in LCLs derived from ADNP patients and sex-age-matched control subjects followed by western blot detection of p62 and LC3 (Additional file 11: Data S9). Prior to Bafilomycin A1 treatment, we detected a non-significant increase in levels of p62/SQSTM1 (*p* = 0.38; ns) and LC3 expression (*p* > 0.99; ns) in patient-derived LCLs. Following Bafilomycin A1 treatment, the expression of p62 (*p* = 0.18; ns) increased in the ADNP deficient cell lines, although non-significant. However, the levels of LC3 (*p* < 0.0001; ****) showed a remarkable increase in the ADNP-patient cell lines, confirming an increased autophagic flux. Given the mitophagy gene signature in LCLs from ADNP patients together with downregulated mitochondrial protein functions identified by LFQ-MS in the human ADNP autopsy brain, we subsequently investigated relative changes in mitochondrial activity and subcellular localization in patient-derived *ADNP* fibroblasts compared to two unaffected controls using the MitoTracker® Red CM-H2XRos probes. Here, we observed a rather faint fluorescence intensity in the ADNP patient fibroblasts compared to the controls. Furthermore, we did not observed a difference in the subcellular localization of the mitochondria in patient fibroblasts as compared to the control subjects (Fig. 9C). Relative MitoTracker® Red CM-H2XRos probe fluorescence per cell, indicative of mitochondrial redox activity, was quantified in patient and control fibroblasts using the Tecan Spark™ and normalized to the brightfield cell count. The mitochondria of patient-derived skin fibroblasts showed a remarkable decrease (*p* = 0.01; *) in fluorescent intensity compared to the control cells, indicating aberrant mitochondrial activity (Fig. 9D). To rule out the possible decrease in the mitochondrial copy number in ADNP LCLs and skin fibroblasts, we determined the mtDNA/nDNA ratio (tRNAleu/B2M) by RT-PCR. Since we could not detect a significant difference in the number of mitochondria in ADNP cell lines compared to controls (Additional file 12: Data S10), we next investigated mitochondrial respiration using the Seahorse analyzer and further addressed the observed mitochondrial dysfunction in patient-derived (blue) and control (red) fibroblasts using the Cell Mito Stress Test to measure changes in oxygen consumption rate (OCR) before/after administration of specific compounds that sequentially affect the different complexes of the mitochondrial respiratory chain. The changes in OCR allowed quantification of several aspects of the mitochondrial respiration. Measurements of the basal respiration showed a significant decrease (*p* = 0.04; *) in the patient fibroblasts compared to the control lines, confirming a reduced activity measured in the fluorescent mitochondrial staining. Besides, we also observed decreased values of proton leak (*p* = 0.21; ns), ATP-linked respiration (*p* = 0.06; ns) and maximal respiratory capacity (*p* = 0.28; ns) in the ADNP cell lines, although the difference was not significant. Lastly, we observed no difference in the spare respiratory capacity (*p* = 0.84; ns) and non-mitochondrial respiration (*p* = 0.79; ns) in patient or control lines (Fig. 9E, F).

## Discussion

In this study, we investigated the cerebellum of a unique six-year-old male child heterozygous for the *ADNP *de novo mutation c.1676dupA/p.His559Glnfs*3 who died of multiple organ failure after a second liver transplant to unravel functional biochemical consequences of the mutation in the human brain.

*ADNP* gene mutations typically result in a syndromic form of autism, co-morbid with ID, termed Helsmoortel–Van der Aa syndrome [2]. The unique autopsy case analyzed in this report presented with moderate ID, whereas developmental milestones such as sitting up and walking independently, language and bladder training were delayed as reported for almost all children of the Helsmoortel–Van der Aa syndrome. With 83.3% of all patients presenting with gastrointestinal problems, our autopsy case presented with parent-reported feeding and eating problems. He also presented with severe autism typified by avoidant behaviors towards other children as reported in almost 93% of patients. To a lesser extent, the child showed generalized symptomatic epilepsy, which was only reported at low frequency in the population (16.2%). Phenotypically, he presented with key features of the syndrome indicated by a prominent forehead, wide nasal bridge, large mouth, widely spaced teeth, and malformed ears as observed for more than the majority of patients. Symptoms were also correlated to urogenital problems (28%), sleep problems (65.2%), and early teething (71.1%) [4, 60]. Clinically, our autopsy patient is thus a bona fide representative of the Helsmoortel–Van der Aa syndrome patient group.

While we have been unable to detect (mutant) ADNP by Western blotting in the autopsy, we do show here a human cerebellar DNA methylation pattern consistent with methylation patterns found in the blood of patients and a transcriptomic profile that bears a significant overlap with the transcriptomic profile of patient cell lines. Proteome analysis of the post-mortem cerebellum also pointed to pathways that have been implicated in the Helsmoortel–Van der Aa syndrome and lead to the identification of ADNP regulatory functions of mitochondria in the human brain. We thus conclude that the ADNP post-mortem cerebellum is valid to study the disorder and further enabled us to confirm earlier observations made in cellular systems and in disease-relevant brain tissue to unravel novel pathways.

### The ADNP brain-specific episignature

ADNP-specific defects in chromatin remodeling translate into genome-wide changes in DNA methylation, including differential methylation of various genes involved in cytoskeletal functions, synaptic transmission, nervous system development [2, 8], calcium-binding [61, 62], and WNT signaling [14], in part resembling a Class I type episignature of Helsmoortel–Van der Aa syndrome [18, 19]. Brain-specific *MAGI2* and *CTNND2* hypomethylation, similar to hypomethylation detected in peripheral blood of ADNP patients, was confirmed by targeted bisulfite pyrosequencing and illustrates partially conserved ADNP-specific episignatures across brain-blood cell types. Interestingly, *CTNND2* dysregulation has been reported in several cases of autism and intellectually disabled patients presenting with behavioral problems and dysmorphic features [63, 64], involving changes in autophagy signaling and arborization of the developing dendrites [65]. Interestingly, our transcription factor (TF) motif enrichment analysis further identified *ADNP* as the main transcription factor controlling the hypomethylated gene cluster, confirming its central regulatory function during brain development [2, 3, 8].

### The *ADNP* mutation affects lineage specification

ADNP regulates genes participating in embryonic development such as *Pax6, Olig2, Sox1, Nestin,* and *Otx2* [8, 39]. Our TF analysis showed enrichment of these lineage specifying genes in the autopsy brain. *Pax6* is important for neuronal development [8, 66], especially the eye, which could potentially be related to the visual problems observed in our patient cohort (73.6%) [4]. *Olig2* is involved in cell fate of ventral neuroectodermal progenitors and differential expression impaired interneuron differentiation, causing cognitive impairments [67]. *Sox1* and its isoforms regulate embryonic development, male sex determination, and cell fate decisions by acting as an inhibitor of neural differentiation [68]. *Nestin* encodes a cytoskeletal protein that is expressed in nerve cells with disturbances causing deficits in motor coordination [69], a hallmark of the Helsmoortel–Van der Aa syndrome [4]. The transcription factor *Otx2* is involved in the differentiation and proliferation of neuronal progenitor cells and as such affecting brain development, craniofacial and sensory organs, and synaptic plasticity [70, 71], which all have been reported to be dysregulated in an *Adnp* heterozygous knockout and CRISPR/Cas9 mouse models [26, 55]. Interestingly, *OTX2* hypermethylation was also confirmed by bisulfite pyrosequencing in the ADNP autopsy brain, correlating with abnormal synaptic plasticity, which has previously been demonstrated by immunohistochemical stainings of PSD95 and NMDAR1 in the hippocampal hillus and dentate gyrus of this patient [72]. Taken together, a mutation in the *ADNP* gene affects brain methylation and expression of genes involved in brain development, neuronal plasticity, and lineage specification.

### The *ADNP* mutation affects WNT signaling

Consistent with the ADNP-specific episignature pathway enrichment outcome, brain transcriptome changes affected similar pathways, involving downregulation of the WNT signaling pathway [14], glutamatergic synaptic transmission [73], cardiac muscle functioning [9] and nervous system development [8]. More particularly, we observed a decrease of β-catenin levels, the major transcriptional driver of the WNT signaling pathway, resulting in the downregulation of neuroectoderm developmental genes and defective neurogenesis [14]. We also found molecular indications for the downregulation of WNT signaling member 10A (WNT10A), a gene essential for tooth morphogenesis [74]. Interestingly, 71% of children present with premature primary tooth eruptions in the Helsmoortel–Van der Aa syndrome [4, 60]. These results were confirmed using bulk mRNA sequencing results of LCLs obtained from several ADNP children showing decreased WNT signaling [17, 60, 72], together with pathways such as Notch [14] and Hedgehog signaling [17] affecting embryogenesis and morphogenesis. Another downregulated gene is the RNA-methylating enzyme *METTL3*, previously reported to be regulated by the β-catenin/WNT signaling pathway in an autism mouse model [75], as well as by the *FMR1* gene, causative of the autistic Fragile X syndrome [76].

### ADNP plays a suggested role in autophagy and aging

We also observed abnormalities in the autophagy pathway, affecting brain homeostasis [77, 78], via downregulation of the autophagy inducer *BECN1* in both brain patient brain tissue and brain samples of *Adnp* deficient mice [24] as well as in post-mortem schizophrenia brains [21]. Furthermore, we also demonstrated elevated LC3 levels in the autopsy brain and LCLs after Bafilomycin A treatment. The reduction in BECN1 levels with the spontaneous increase in LC3 protein levels might reflect a compensatory mechanism [79]. In addition, ADNP was also shown to bind LC3 directly in a human neuroblastoma cell line, and its association was increased in the presence of the NAP (Davunetide) octapeptide [78]. Furthermore, we observed expression changes in members of the nonsense-mediated decay (NMD) pathway, which trigger the autophagy process [80]. In fact, changes in mRNA levels of the NMD-members *SMG5* and *UPF3B* have been associated with intellectual syndromic and non-syndromic intellectual disability, autism, childhood onset schizophrenia and ADHD [81–83]. Interestingly, the *Slc12a2* and *Slc9a3* family members were shown to be regulated in an age-dependent manner in the hippocampus, cortex, and spleen of *Adnp* haploinsufficient mice [25], and early-onset hippocampal tauopathy, a marker for aging and neurodegeneration, was reported in this young subject by an independent study [72]. *RUBCN* is a negative regulator of autophagy which also correlates with aging [84].

### HP1beta involvement

Intriguingly, we also detected a significant increase in protein levels of the repressive chromatin ADNP-interactor HP1β in our proteomics experiment exclusively, which is consistent with as yet unpublished observations in the brain of our novel *Adnp* frameshift mouse model [7], again highlighting the resemblance between murine and human data and the effects that mutations in *ADNP* have on the expression of its direct interaction partners [9].

### Novel ADNP interactions in the brain: immune signature, the cytoskeleton and mitochondrial involvement

In our study, pathways involving cytoskeleton dynamics [85], T-helper cell differentiation and immune-associated pathways [86] were found to be transcriptionally upregulated. However, it cannot be excluded that the observed immune system malfunctions could be related to the reported multiple organ failure in the patient. Moreover, a substantial part of Helsmoortel–Van der Aa patients presents with (pharmacologically treated) thyroid hormone problems or epilepsy, which may also affect inflammation-related processes [4]. Upon further downstream protein expression profiling of the ADNP patient versus healthy cerebellum proteome by mass spectrometry analysis, differentially expressed proteins showed enrichment for mitochondrial dysfunction. Mitochondrial dysfunction has been linked to the onset of autism [87, 88] and epilepsy [89, 90] by multiple studies, both key features present in the clinical presentation of the current case. Besides, we also observed significant downregulation of cytoskeletal functions and sirtuin signaling. Of special note, ADNP associates with cytoskeletal microtubules through its SxIP motif and the microtubule end binding proteins 1 and 3 (EB1/EB3) in differentiated neurons [22] with microtubule deficits of human ADNP mutants in cell cultures (e.g., p.Ser404*, p.Tyr719*, and p.Arg730*) [72, 91, 92]. Interestingly, sirtuins (SIRTs) are vital NAD^+^-dependent deacetylase enzymes that regulate autophagy [93], mitophagy [94], aging [95], and cytoskeletal (microtubule) functions [96] via dynamic changes in protein acetylation [54]. Similar to ADNP, SIRT1 regulates autophagy and mitochondrial functions [49], maintains genomic stability [45], enhances synaptic plasticity [46], suppresses inflammation [47], regulates cellular aging [48] and promotes neuroprotection [97]. Only recently, ADNP-SIRT1 interactions could be detected via WRD5 in a human neuroblastoma cell line [16], which could now be further validated in murine cerebellum modeling the died ADNP patient. Besides, we showed biochemical evidence for indirect binding of ADNP and SIRT1 via the microtubule end binding proteins (EB1/EB3), further linking autophagy, mitophagy, and cytoskeletal (microtubule) brain functions. Similarly, in other neurodevelopmental disorders such as Koolen-De Vries syndrome, dysfunctional autophagy was found to cause synaptic deficits in human IPSC cultures [79, 98]. Our findings were further supported by gene set enrichment analysis of cellular stress responses [34] and mitochondrial activity-based assays which reveal disrupted mitochondrial gene expression via autophagy (mitophagy) processes and decreased mitochondrial activity in patient-derived fibroblasts and LCLs with an *ADNP* mutation. Interestingly, the ADNP-derived drug NAP (Davunetide) was shown to improve microtubule-dependent traffic, restore the autophagic flux and potentiate autophagosome-lysosome fusion, leading to autophagic vacuole clearance in Parkinson’s disease cells [99]. Finally, protein–protein interaction network analysis of our differential proteome analysis predicted SIRT1 in a central hub which links chromatin remodelers (e.g., ADNP, SMARCC2, HDAC2 and YY1) with autophagy signaling (BECN1, LAMP1 and LC3). Various chromatin remodeling proteins of the network have previously been ascribed as part of the ADNP-WDR5-SIRT1-BRG1-HDAC2-YY1 chromatin complex, which in part shares co-expression via transcriptional regulation [16].

In conclusion, our integrative multi-omics study for the first time has confirmed various ADNP mutant associated neurodevelopmental affected pathways at the epigenome-transcriptome-proteome level in primary brain tissue of an ADNP child, which previously have been described in in vitro LCL cultures and in vivo animal experiments substantiating strong cross-species and cross-cell type of molecular ADNP (disease) features. Moreover, our results hint towards a new functional link between the chromatin remodeler ADNP and the NAD^+^-deacetylase SIRT1 to control cytoskeletal and mitochondrial autophagy stress responses in neurodevelopment and plasticity. This novel molecular mechanism holds promise for new therapeutic strategies aimed at restoring mitochondrial (dys)function(s) in the Helsmoortel–Van der Aa syndrome.

## Limitations of the study

In this study, we performed a molecular and biochemical autopsy study on the cerebellar tissue of a patient with Helsmoortel–Van der Aa syndrome, who has died after multiple organ failure following a second liver transplantation. We acknowledge several limitations that are either intrinsic to the study design or linked to the experimental course of action. First, we report the only post-mortem brain tissue that is currently available and compare it with an age-matched control subject. We acknowledge a gender difference between the ADNP patient (male) and control subject (female) but have consistently taken this difference into account during all bioinformatic analyses. In addition, we acknowledge that our control subject has a clinical diagnosis of Rett syndrome. However, we sequenced the entire *ADNP* gene, showing no genetic defect in the specimen. In addition, an expert pathologist examined brain sections using different immunohistochemical techniques, resulting no morphological abnormalities in the cerebellum. Although we are aware of the unique value of this material, we do acknowledge the lack of statistical power due to the limited sample size (n = 1). To overcome the lack of statistical power, we compared our findings of the human autopsy brain to several primary cell lines (e.g., LCLs and skin fibroblasts) derived from patients with different *ADNP* mutations and looked for gene expression similarities and dissimilarities across these tissues. Here, we observed a similar trend of gene expression changes in the human brain with in vitro and in vivo model systems, indicating a strong conservation of the *ADNP* gene and functioning. However, we report that this mutation is unique on its own, and no patient has been diagnosed with this *ADNP* mutation before. Therefore, comparisons to individuals with different *ADNP* mutations or genetically engineered mouse models cannot completely represent the molecular mechanisms underlying this specific mutation. On the topic of preservation of the autopsy material, we recognize a long post-mortem interval time i.e., the period of death of the patient and the time of the brain biopsy, which was 35-h after which liquid nitrogen was applied. To examine DNA and mRNA preservation in the cerebellum of our autopsy case, we subjected the extracted materials to the most sensitive bioanalyzer technologies to control for proper integrity. In addition, we also implemented very extensive validations e.g., pyrosequencing and RT-PCR for the implemented omics techniques using adequate controls. Similar to mRNA integrity, the preservation of proteins is not homogeneous since some molecules are more vulnerable than others [100]. In particular, we were unable to detect ADNP levels by means of western blotting in the cerebellum, but we did detect the protein in the cerebellum of the control subject. Since ADNP is already an unstable protein [7, 29], the absence of the protein could potentially be attributed to degradation by active proteases in the brain during the post-mortem interval [101]. Lastly, we noted prominent changes in immune system-related pathways as indicated by our methylation, transcriptome, and proteome analyses of the autopsy brain. Although a subset of patients presented with thyroid problems [2] and recent investigations indicated a role of ADNP in T-helper cell differentiation [86], we show a critical attitude towards the interpretation of these results, as we cannot rule out certain bias in gene expression alterations following two liver transplantations and the administration of multiple antiepileptic drugs during the lifespan of this child with an *ADNP* mutation.

## Supplementary Information

**Additional file 1**: **Table S1**. Specifications of the human subjects, lymphoblastoid and fibroblastic cell lines. The table represents the anonymized patient IDs as a fictive number together with the WES-validated mutation in the ADNP gene. RNA purity determined by the 260/280 ratio and RIN integrity score of the RNA samples were also reported. **Table S2**. Tested antibody overview. The table indicates the used antibodies for this study together with the manufacture and catalog number, host species, predicted reactivity, peptide sequence and the optimized dilution for each western blot experiment. **Table S3**. Pyrosequencing primers. Table contains gene name, forward primer (5’ 3’), reverse primer (5’ 3’), location of the biotin tag of either the forward (Fwd) or reverse (Rev) primer, sequencing primer (5’ 3’), nucleotide sequence for CpG analysis and predication of the EPIC Beadchip array result. **Table S4**. RT-PCR primer sequences. The table represent the forward and reverse primer sequences (5’ 3’) for expression analysis of ADNP, brain and lymphoblastoid transcriptome, and mitochondrial gene panel confirmations. **Table S5**. Mitophagy-related gene panel using for screening the RNA sequencing data of ADNP lymphoblastoid cell lines (LCLs). **Table S6**. Phenotype and clinical features of the post-mortem ADNP patient carrying the c.1676duplA/p.His559Glnfs*3 mutation in comparison with a cohort study by Van Dijck et al. (2019) entailing 78 HVDAS individuals.

**Additional file 2**: **Human Infinium EPIC BeadChip Array data of the ADNP cerebellum as compared to an age-matched control subject**. CpG probes with an absolute β-value > 0.1 (10% methylation differences) are represented.

**Additional file 3**: **Transcription factor motif analysis with Irregulon**. Enriched transcription factor motifs in the ADNP cerebellum using the identified hypo- and hypermethylated genes. Motif enrichment was scored using the normalized enrichment score (NES).

**Additional file 4**: List of differentially expressed genes in the ADNP cerebellum as compared to an age-matched control subject using the NOIseq non-parametric analysis package.

**Additional file 5**: List of differentially expressed genes in the ADNP patient-derived lymphoblastoid cell lines as compared to age- and sex-matched control lymphoblastoid cell lines using the DESeq2 analysis package.

**Additional file 6**: List of overlapping differentially expressed genes in the ADNP cerebellum that overlap with the differentially expressed genes in the ADNP patient-derived lymphoblastoid cell lines.

**Additional file 7**: **Correlation heatmap of the label-free quantification mass spectrometry (LFQ-MS) experiment in the ADNP cerebellum**. Pairwise correlations of protein abundances characterizing the relationships between proteins of the global mass spectrometry experiment. Correlations between all proteins, clustering of protein groups and similarly behaving proteins are represented in the protein-protein correlation matrix calculated on the logarithmic intensities. A strong correlation between technical replicates (n = 5) was observed (red color), indicating high reproducibility as tested by the Pearson correlation. Negative correlations are represented in a blue color (see scaled bar legend).

**Additional file 8**: List of differentially expressed proteins in the ADNP cerebellum (LFQ-MS).

**Additional file 9**: **Mitophagy transcriptomic gene signature in the ADNP cerebellum**. Expression levels of mitophagy-related genes in the ADNP patient cerebellum compared to an age-matched control subject. mRNA sequencing demonstrated both an upregulated (red) as well as a downregulated (blue) mitophagy gene signature in the ADNP patient cerebellum as compared to an age-matched control subject.

**Additional file 10**: **Autophagic flux assessment in ADNP patient-derived lymphoblastoid cell lines using Bafilomycin A1**. **A**. The autophagic flux was determined in ADNP patient-derived and age- and sex-matched control lymphoblastoid cell lines by treatment with 160 nM of Bafilomycin A1 (BAF) for two hours. Protein extracts of untreated and BAF-treated cells were subjected to western blotting using anti-p62/SQSTM1 and anti-LC3 antibodies to assess the autophagic flux. Although p62 expression increased after BAF treatment (+BAF) compared to untreated (-BAF) cells, the difference was not significant in patients (PAT) and controls (CTR). However, the expression of LC3 significantly increased after BAF treatment and was significantly increased in PAT versus CTR post-treatment compared to untreated patient cells (PAT-BAF). All western blots were controlled by GAPDH to ensure equal loading. Image quantification was performed using ImageJ software. **B**. Graphical representation was performed in GraphPad Prism version 9.3.1 using a 2-way ANOVA with Sidak’s multiple comparisons test to assess the interaction of the genotype (PAT versus CTR) and treatment (-BAF versus +BAF).

**Additional file 11**: Determination of the mitochondrial DNA copy number (mtDNA-CN) in ADNP patient-derived lymphoblastoid cells and fibroblastic cell lines.

**Additional file 12**: Raw western blotting images.

## Data Availability

The data presented in this study is available in the article or contained in the supplementary information. Correspondence and requests for material/raw data should be addressed to Claudio Peter D’Incal.

## Declarations

### Accordance to ARRIVE guidelines

We confirm that this study is reported in accordance with the ARRIVE guidelines (https://arriveguidelines.org).
