# Correction to: ADNP dysregulates methylation and mitochondrial gene expression in the cerebellum of a Helsmoortel–Van Der Aa syndrome autopsy case

**DOI:** 10.1186/s40478-024-01787-y

**Published:** 2024-10-24

**Authors:** Claudio D’Incal, Anke Van Dijck, Joe Ibrahim, Kevin De Man, Lina Bastini, Anthony Konings, Ellen Elinck, Claudia Theys, Illana Gozes, Zlatko Marusic, Mirna Anicic, Jurica Vukovic, Nathalie Van der Aa, Ligia Mateiu, Wim Vanden Berghe, R. Frank Kooy

**Affiliations:** 1 Department of Medical Genetics, University of Antwerp, Prins Boudewijnlaan 43/6, 2650 Edegem, Antwerp, Belgium; 2 Protein Chemistry, Proteomics and Epigenetic Signaling (PPES), Epigenetic Signaling lab (PPES), Department of Biomedical Sciences, University of Antwerp, Universiteitsplein 1, 2610 Wilrijk, Antwerp, Belgium; 3 Family Medicine and Population Health (FAMPOP), Department of Medicine and Health Sciences, University of Antwerp, Antwerp, Belgium; 4 The Elton Laboratory for Molecular Neuroendocrinology, Department of Human Molecular Genetics and Biochemistry, Faculty of Medical & Health Sciences, Adams Super Center for Brain Studies and Sagol School of Neuroscience, Tel Aviv University, Tel Aviv, Israel; 5 Clinical Department of Pathology and Cytology, University Hospital Centre Zagreb, Zagreb, Croatia; 6 Division of Pediatric Gastroenterology, Hepatology and Nutrition, Department of Pediatrics, School of Medicine, University Hospital Centre Zagreb, Zagreb, Croatia

Correction to: ***acta neuropathol commun *12**, 62 (2024)

10.1186/s40478-024-01743-w

Following publication of the original article [1], the authors identified three errors. An error in the author name of Illana Gozes. The first letter of the given name contained an error. The author Claudia Theys was omitted from the author group. Affiliation 4 was partly incorrect.

The incorrect author name is: lllana Gozes

The correct author name is: Illana Gozes

The Author contributions section currently reads:

**Author contributions**

C.P.D. performed the experiments, conceptualized the experimental design, performed data analysis, wrote the manuscript text, and prepared all the figures. A.V.D. is the neurologist who collected the clinical data. J.I. provided the bioinformatic analysis of the methylation data. K.D.M., L.B., and A.K. were active participants in the experiments under supervision of C.D., E.E. conducted all the gene expression assays using RT-PCR under supervision of C.D., I.G. was involved in the tissue collection of the autopsy and reviewed the manuscript. Z.M. provided the postmortem tissue. M.A. and J.V. also provided the clinical data together with A.V.D., N.V.D.A. is a pediatrician and responsible for obtaining the LCLs from her patients. L.M. provided the bioinformatic analysis of the RNA sequencing of the brain and LCLs. W.V.B. reviewed the manuscript. R.F.K. reviewed the manuscript. All authors reviewed the manuscript.

The Author contributions section should read:

**Author contributions**

C.P.D. performed the experiments, conceptualized the experimental design, performed data analysis, wrote the manuscript text, and prepared all the figures. A.V.D. is the neurologist who collected the clinical data. J.I. provided the bioinformatic analysis of the methylation data. K.D.M., L.B., and A.K. were active participants in the experiments under supervision of C.D., E.E. conducted all the gene expression assays using RT-PCR under supervision of C.D., C.T. assisted with the functional mitochondrial analysis, I.G. was involved in the tissue collection of the autopsy and reviewed the manuscript. Z.M. provided the postmortem tissue. M.A. and J.V. also provided the clinical data together with A.V.D., N.V.D.A. is a pediatrician and responsible for obtaining the LCLs from her patients. L.M. provided the bioinformatic analysis of the RNA sequencing of the brain and LCLs. W.V.B. reviewed the manuscript. R.F.K. reviewed the manuscript. All authors reviewed the manuscript.

The affiliation 4 currently reads:

4 The Elton Laboratory for Molecular Neuroendocrinology, Department of Human Molecular Genetics and Biochemistry, Sackler Faculty of Medicine, Adams Super Center for Brain Studies and Sagol School of Neuroscience, Tel Aviv University, Tel Aviv, Israel

The affiliation 4 should read:

4 The Elton Laboratory for Molecular Neuroendocrinology, Department of Human Molecular Genetics and Biochemistry, Faculty of Medical & Health Sciences, Adams Super Center for Brain Studies and Sagol School of Neuroscience, Tel Aviv University, Tel Aviv, Israel

The author group and affiliations have been updated above and the original article [1] has been corrected.
