# Supplementary material for: ADNP dysregulates methylation and mitochondrial gene expression in the cerebellum of a Helsmoortel–Van der Aa syndrome autopsy case
Source: Acta Neuropathol Commun. 2024 Apr 18;12:62. doi: 10.1186/s40478-024-01743-w (PMC11027339; doi:10.1186/s40478-024-01743-w)
Supplement: Supplementary file 1 — Additional file 1: Table S1. Specifications of the human subjects, lymphoblastoid and fibroblastic cell lines. The table represents the anonymized patient IDs as a fictive number together with the WES-validated mutation in the ADNP gene. RNA purity determined by the 260/280 ratio and RIN integrity score of the RNA samples were also reported. Table S2. Tested antibody overview. The table indicates the used antibodies for this study together with the manufacture and catalog number, host species, predicted reactivity, peptide sequence and the optimized dilution for each western blot experiment. Table S3. Pyrosequencing primers. Table contains gene name, forward primer (5’ 3’), reverse primer (5’ 3’), location of the biotin tag of either the forward (Fwd) or reverse (Rev) primer, sequencing primer (5’ 3’), nucleotide sequence for CpG analysis and predication of the EPIC Beadchip array result. Table S4. RT-PCR primer sequences. The table represent the forward and reverse primer sequences (5’ 3’) for expression analysis of ADNP, brain and lymphoblastoid transcriptome, and mitochondrial gene panel confirmations. Table S5. Mitophagy-related gene panel using for screening the RNA sequencing data of ADNP lymphoblastoid cell lines (LCLs). Table S6. Phenotype and clinical features of the post-mortem ADNP patient carrying the c.1676duplA/p.His559Glnfs*3 mutation in comparison with a cohort study by Van Dijck et al. (2019) entailing 78 HVDAS individuals. [file 40478_2024_1743_MOESM1_ESM.docx]

**Supplementary tables**

**Additional file 1: Table S1. Specifications of the human subjects, lymphoblastoid and fibroblastic cell lines.** The table represents the anonymized patient IDs as a fictive number together with the WES-validated mutation in the ADNP gene. RNA purity determined by the 260/280 ratio and RIN integrity score of the RNA samples were also reported.

| **Patient ID** | ***De novo* *ADNP* mutation** | **Mutational class** | **Involved experiment** | **260/280 ratio** | **RIN Score** |
| --- | --- | --- | --- | --- | --- |
| TR1399 | No *ADNP* mutation | No mutation, CTR subject | RNA sequencing | 2.0 | 8.9 |
| TR1400 | No *ADNP* mutation | No mutation, CTR subject | RNA sequencing | 2.0 | 9.5 |
| TR1402 | No *ADNP* mutation | No mutation, CTR subject | RNA sequencing; mitochondrial assays | 2.0 | 9.4 |
| TR1422 | No *ADNP* mutation | No mutation, CTR subject | RNA sequencing; mitochondrial assays | 2.0 | 8.8 |
| TR1679 | p.Gln40* | N-terminal mutation | RNA sequencing | 2.0 | 10 |
| TR1633 | p.Ser404* | Perinuclear (NLS destructive) mutation | RNA sequencing | 2.0 | 10 |
| TR1647 | p.Leu831Ilefs*82 | C-terminal mutation | RNA sequencing | 2.0 | 9.8 |
| TR1648 | p.Leu831Ilefs*82 | C-terminal mutation | RNA sequencing; mitochondrial assays | 2.1 | 10 |
| TR1626 | p.Asn832Lysfs*81 | C-terminal mutation | RNA sequencing | 2.1 | 10 |
| TR1548 | p.Asn832Lysfs*81 | C-terminal mutation | RNA sequencing; mitochondrial assays | 2.0 | 10 |
| CTR cerebellum | No *ADNP* mutation | No mutation, CTR subject | RNA sequencing | 2.0 | 6.5 |
| ADNP patient cerebellum | p.His559Glnfs*3 | Perinuclear (NLS destructive) mutation | RNA sequencing | 2.0 | 6.5 |

**Additional file 1: Table S2.Tested antibody overview**. The table indicates the used antibodies for this study together with the manufacture and catalog number, host species, predicted reactivity, peptide sequence and the optimized dilution for each western blot experiment.

| **Antibody** | **Product number** | **Host species** | **Reactivity** | **Immunogen sequence** | **Dilution** |
| --- | --- | --- | --- | --- | --- |
| *ADNP Polyclonal*  *antibody* | Aviva Systems;  ARP39186_P050 | Rabbit | Mouse, rat, and human | N-terminal antibody  aa 35-82 | 1:1000 in 5% NFDM/TBST |
| *ADNP monoclonal*  *antibody* | Abcam; ab300114 | Rabbit | Mouse, rat, and human | C-terminal antibody  aa 850-1050 | 1:1000 in 5% NFDM/TBST |
| *SIRT1 Rabbit monoclonal antibody* | Abcam; ab189494 | Rabbit | Mouse, rat, and human | Proprietary to Abcam | 1:1000 in 5% NFDM/TBST |
| *CBX1/HP1β monoclonal antibody* | Cell Signaling Technology; 8676T | Rabbit | Mouse, rat, and human | Synthetic peptide corresponding to residues surrounding Gln69 of human HP1β protein | 1:1000 in 5% NFDM/TBST |
| *LC3-I/II monoclonal antibody* | NanoTools; 0231-100/LC3-5F10 | Mouse | Human, mouse, rat,  dog, hamster | N-terminus of LC3-B | 1:1000 in 5% NFDM/TBST |
| *BECN1 monoclonal antibody* | BD Transduction Laboratories™; 612112 | Mouse | Mouse, Rat, Dog, Chicken | Human Beclin aa. 171-291 | 1:1000 in 5% NFDM/TBST |
| *β-Catenin monoclonal antibody* | Cell Signaling Technology; 8480 | Rabbit | Mouse, rat, and human | Synthetic peptide corresponding to residues surrounding Pro714 of human β-catenin | 1:1000 in 5% NFDM/TBST |
| *MAPRE1/EB1 monoclonal antibody* | Abcam; ab53358 | Rat | Human, mouse, hamster | Synthetic peptide. This information is proprietary to Abcam and/or its suppliers. | 1:1000 in 5% NFDM/TBST |
| *EB3 monoclonal antibody* | Abcam; ab157217 | Rabbit | Human, rat | Synthetic peptide within Human EB3. The exact sequence is proprietary. | 1:1000 in 5% NFDM/TBST |

**Additional file 1: Table S3. Pyrosequencing primers**. Table contains gene name, forward primer (5’ 🡪 3’), reverse primer (5’ 🡪 3’), location of the biotin tag of either the forward (Fwd) or reverse (Rev) primer, sequencing primer (5’ 🡪 3’), nucleotide sequence for CpG analysis and predication of the EPIC Beadchip array result.

| *Gene name* | *Forward primer* | *Reverse primer* | *Biotin tag* | *Sequencing primer* | *Sequence to analyze* | *EPIC prediction* |
| --- | --- | --- | --- | --- | --- | --- |
| *OTX2* | GTTTAGTTTTTAGGGAGATTTGTTGAGAA | CAACCACCTATTTTCCTCCCCCTATTAT | *Fwd* | CTCTCCCTACCATTAA | TTAAAAAATTTACRTCAAAAAATTACCAAA AAAAC | *Hypermethylated* |
| *SLC25A21* | GGAGTTTTTATTATTTGTTGGGGATTT | *AAAACAAATATTCCCATATATTAACCTACC* | *Rev* | *ATTTGTTGGGGATTTG* | *TTTATYGTTATTTTATGGGTATATTGTAGGTTTTTT* | *Hypermethylated* |
| *DNAJ6* | *AGGGAATTGGTTTTATGGTAATATTTAGTA* | *TCTTTACCTCTAAAATCTCTATACCCATAT* | *Rev* | *TGTTTTTTAATTTTGTGATTAAGG* | *TTATAAAGGYGTTAGAAAAGTATTGTTATT ATTATTTTT* | *Hypermethylated* |
| *COL4A2* | *GTTATTGGGGTAAATGTTGTTGTAGTTATG* | *CCCCTATCTCTCTATAATACCCACTA* | *Rev* | *TTTGTTAGTTTTAATTTAGAGAA* | *AGATTTATTGAAAYGTTATGGGTAAAAGAGATTTTTTTAAAAT* | *Hypomethylated* |
| *MAGI2* | *GATATTTGGGGATGTTAAATGTAGTTGAT* | *CTTTCTCCTATATTATACCCTACTAATCT* | *Rev* | *AAATGTAGTTGATTGATATAATTG* | *TTAGATTTTAYGATTTAGGGTTTTATTAGT* | *Hypomethylated* |
| *CTNND2* | *GGGTGATGTTGTAGTATAAAGTTTG* | *CCCCAAAATCAAAATTCTCCTC* | *Rev* | *TGTGGGATAGGTTAGT* | *AGGTYGGGAAYGTAAATAGGGTATTTATGT TGTAGT* | *Hypomethylated* |

**Additional file 1: Table S4. RT-PCR primer sequences**. The table represent the forward and reverse primer sequences (5’ 🡪 3’) for expression analysis of ADNP, brain and lymphoblastoid transcriptome, and mitochondrial gene panel confirmations.

| ***Target gene*** | | **Name** | **Forward primer** | **Reverse primer** |
| --- | --- | --- | --- | --- |
| **ADNP expression analysis** | | | | |
| ***ADNP (duplication)*** | | Activity-dependent Neuroprotective Protein | AGCTGAGAAAGTCCCAGCAG | TGGGTCCTTTTAGGATTGAAAA |
| ***ADNP (5’ end of exon 5)*** | | Activity-dependent Neuroprotective Protein | CACCTTGCATGGTAGCCTTT | TGAGGTTGACCAAGACGATG |
| ***Gene*** | **Name** | | **Forward primer** | **Reverse primer** |
| **Brain transcriptome** | | | | |
| ***METTL3*** | N6-Adenosine-Methyltransferase | | GTGTCGGAGGTGATTCCAGT | CTGCGCATCTCATCATCTGT |
| ***BECN1*** | Beclin 1 | | GGCCAATAAGATGGGTCTGA | TGTCTCGCCTTTCTCAACCT |
| ***CTNNB1*** | Catenin beta 1 | | GAAACGGCTTTCAGTTGAGC | CTGGCCATATCCACCAGAGT |
| **Lymphoblastoid transcriptome** | | | | |
| ***CBX3*** | Chromobox 3 (heterochromatin protein 1 beta) | | TTGGCAGTTTAGGACCTGCT | TGTTCTTCCTGGCTTTTGCT |
| ***WNT10A*** | Wnt family member 10 A | | GGTTGCTCCACACCCTAAAA | ATGATGAAGGGAATGGTGGA |
| ***CTNNAl1*** | Catenin alpha 1 | | AAAGCCAGACAAGCCTGACTCT | AGCAAACCCAGCTTAAGTCCAA |
| ***SMG5*** | SMG5 Nonsense Mediated MRNA Decay Factor | | TCAAGCGGCTGTATGACAAG | CTGCTTTTGGGCTGTAGGAG |
| ***UPF3B*** | UPF3B regulator of nonsense mediated mRNA decay | | CTTCAGGGCAAAGAATAGAGAGA | TTGACACAAGACTTACTCCTCTG |
| **Meta-analysis of brain versus lymphoblastoid transcriptomes** | | | | |
| ***IGFBP2*** | Insulin like growth factor binding protein 2 | | CCTCAAGTCGGGTATGAAGG | ACCTGGTCCAGTTCCTGTTG |
| ***WNT2*** | Wnt family member 2 | | GTGGATGCAAAGGAAAGGAA | AGCCAGCATGTCCTGAGAGT |
| ***SLC25A25*** | Solute carrier family 25 member 25 | | GTCCCGGATGAGTTCACAGT | CATCTGAGTGAAGCCACCAA |
| ***RUBCN*** | Rubicon autophagy regulator | | GCCATGTGAAAGACTGCTGA | AAGCTTTGGCTTCCTGTGAA |
| ***RUNX1*** | RUNX family transcription factor 1 | | GAGGGTCACTCCAAGGATCA | TGGGCAGAAATCAAATCCTC |
| ***METTL3*** | N6-Adenosine-Methyltransferase | | GTGTCGGAGGTGATTCCAGT | CTGCGCATCTCATCATCTGT |
| ***BMP6*** | Bone morphogenetic protein 6 | | GCGACACCACAAAGAGTTCA | CCCATACTACACGGGTGTCC |
| **Mitophagy gene signature in ADNP lymphoblastoid cells** | | | | |
| ***MFN2*** | Mitofusin 2 | | GACCCCGTTACCACAGAAGA | GCAGAACTTTGTCCCAGAGC |
| ***MAPK1*** | Mitogen-Activated Protein Kinase 1 | | CTCCTGTGGTGCAGATGAGA | CAGCACAAGAAAAGGCAACA |
| ***BECN1*** | Beclin 1 | | GGCCAATAAGATGGGTCTGA | TGTCTCGCCTTTCTCAACCT |
| ***MCL1*** | MCL1 Apoptosis Regulator, BCL2 Family Member | | TGCTGGAGTAGGAGCTGGTT | CCTCTTGCCACTTGCTTTTC |
| ***USP15*** | Ubiquitin Specific Peptidase 15 | | AAGGTCAACTCACGGGACAC | GATCCCAATCCAAAGCAAGA |
| ***USP8*** | Ubiquitin Specific Peptidase 8 | | CTCCGGAGTCTGAAAGATGC | CTGGGCAGCAGGTTTAGAAG |
| ***TBK1*** | TANK Binding Kinase 1 | | AGCGGCAGAGTTAGGTGAAA | TGAGTGCCTTCTTGATGTGC |
| ***UBE2N*** | Ubiquitin Conjugating Enzyme E2 N | | TGGTGTCTTGCCACAATGTT | GACTCACAAATGCCTCAGCA |
| ***MTFR2*** | mitochondrial fission regulator 2 | | TGATCCCGCTCTTGAACTCT | GTGACAGTGGATCCCGAACT |

**Additional file 1: Table S5. Mitophagy-related gene panel using for screening the RNA sequencing data of ADNP lymphoblastoid cell lines (LCLs).**

| ***Official Gene symbol*** | ***Description*** | ***Biological Function*** | ***References*** |
| --- | --- | --- | --- |
| ***BNIP3*** | BCL2/adenovirus E1B 19kDa interacting protein 3 | Mitophagy core | J Biol Chem. 2012 Jun 1;287(23):19094-104. Microtubule-associated protein 1 light chain 3 (LC3) interacts with Bnip3 protein to selectively remove endoplasmic reticulum and mitochondria via autophagy. Hanna RA, Quinsay MN, Orogo AM, Giang K, Rikka S, Gustafsson ÅB. |
| ***MAPK1*** | mitogen-activated protein kinase 1 | Mitophagy core | Autophagy. 2015;11(2):332-43.  Mitophagy is primarily due to alternative autophagy and requires the MAPK1 and MAPK14 signaling pathways. Hirota Y, Yamashita S, Kurihara Y, Jin X, Aihara M, Saigusa T, Kang D, Kanki T. |
| ***MAPK14*** | mitogen-activated protein kinase 14 | Mitophagy core | Autophagy. 2015;11(2):332-43. Mitophagy is primarily due to alternative autophagy and requires the MAPK1 and MAPK14 signaling pathways. Hirota Y, Yamashita S, Kurihara Y, Jin X, Aihara M, Saigusa T, Kang D, Kanki T. |
| ***SMURF1*** | SMAD specific E3 ubiquitin protein ligase 1 | Mitophagy core | Nature. 2011 Dec 1;480(7375):113-7. Image-based genome-wide siRNA screen identifies selective autophagy factors. Orvedahl A, Sumpter R Jr, Xiao G, Ng A, Zou Z, Tang Y, Narimatsu M, Gilpin C, Sun Q, Roth M, Forst CV, Wrana JL, Zhang YE, Luby-Phelps K, Xavier RJ, Xie Y, Levine B. |
| ***ATP13A2*** | ATPase type 13A2 | Mitophagy core | Autophagy. 2013 Nov 1;9(11):1828-36. Proteolytic processing of Atg32 by the mitochondrial i-AAA protease Yme1 regulates mitophagy. Wang K, Jin M, Liu X, Klionsky DJ. |
| ***SPATA18*** | spermatogenesis associated 18 | Mitophagy core | PLoS One. 2011 Jan 17;6(1):e16054. Possible existence of lysosome-like organella within mitochondria and its role in mitochondrial quality control. Miyamoto Y, Kitamura N, Nakamura Y, Futamura M, Miyamoto T, Yoshida M, Ono M, Ichinose S, Arakawa H. |
| ***TBK1*** | TANK-binding kinase 1 | Mitophagy core | Proc Natl Acad Sci U S A. 2016 Apr 12;113(15):4039-44.  Phosphorylation of OPTN by TBK1 enhances its binding to Ub chains and promotes selective autophagy of damaged mitochondria. Richter B, Sliter DA, Herhaus L, Stolz A, Wang C, Beli P, Zaffagnini G, Wild P, Martens S, Wagner SA, Youle RJ, Dikic I. |
| ***CERS1*** | ceramide synthase 1 | Mitophagy core | Nat Chem Biol. 2012 Oct;8(10):831-8. Ceramide targets autophagosomes to mitochondria and induces lethal mitophagy. Sentelle RD, Senkal CE, Jiang W, Ponnusamy S, Gencer S, Selvam SP, Ramshesh VK, Peterson YK, Lemasters JJ, Szulc ZM, Bielawski J, Ogretmen B. |
| ***VCP*** | valosin containing protein | Mitophagy core | J Cell Biol. 2010 Dec 27;191(7):1367-80. Proteasome and p97 mediate mitophagy and degradation of mitofusins induced by Parkin. Tanaka A, Cleland MM, Xu S, Narendra DP, Suen DF, Karbowski M, Youle RJ. |
| ***HDAC6*** | histone deacetylase 6 | Mitophagy core | J Cell Biol. 2010 May 17;189(4):671-9. Disease-causing mutations in parkin impair mitochondrial ubiquitination, aggregation, and HDAC6-dependent mitophagy. Lee JY, Nagano Y, Taylor JP, Lim KL, Yao TP. |
| ***USP8*** | ubiquitin specific peptidase 8 | Mitophagy core | Autophagy. 2015 Nov 3;11(2):428-9.  USP8 and PARK2/parkin-mediated mitophagy. Durcan TM, Fon EA. |
| ***PINK1*** | PTEN induced putative kinase 1 | Mitophagy core | Nature. 2015 Aug 20;524(7565):309-314.  The ubiquitin kinase PINK1 recruits autophagy receptors to induce mitophagy. Lazarou M, Sliter DA, Kane LA, Sarraf SA, Wang C, Burman JL, Sideris DP, Fogel AI, Youle RJ. |
| ***MUL1*** | mitochondrial E3 ubiquitin protein ligase 1 | Mitophagy core | Autophagy. 2015;11(8):1216-29.  Mitochondrial outer-membrane E3 ligase MUL1 ubiquitinates ULK1 and regulates selenite-induced mitophagy. Li J, Qi W, Chen G, Feng D, Liu J, Ma B, Zhou C, Mu C, Zhang W, Chen Q, Zhu Y. |
| ***FBXO7*** | F-box protein 7 | Mitophagy core | Nat Neurosci. 2013 Sep;16(9):1257-65.  The Parkinson's disease-linked proteins Fbxo7 and Parkin interact to mediate mitophagy. Burchell VS, Nelson DE, Sanchez-Martinez A, Delgado-Camprubi M, Ivatt RM, Pogson JH, Randle SJ, Wray S, Lewis PA, Houlden H, Abramov AY, Hardy J, Wood NW, Whitworth AJ, Laman H, Plun-Favreau H. |
| ***TBC1D17*** | TBC1 domain family, member 17 | Mitophagy core | Elife. 2014 Feb 25;3:e01612.  Mitochondrial Rab GAPs govern autophagosome biogenesis during mitophagy. Yamano K, Fogel AI, Wang C, van der Bliek AM, Youle RJ. |
| ***TBC1D15*** | TBC1 domain family, member 15 | Mitophagy core | Elife. 2014 Feb 25;3:e01612.  Mitochondrial Rab GAPs govern autophagosome biogenesis during mitophagy. Yamano K, Fogel AI, Wang C, van der Bliek AM, Youle RJ. |
| ***RHEB*** | Ras homolog enriched in brain | Mitophagy core | Cell Metab. 2013 May 7;17(5):719-30.  Rheb regulates mitophagy induced by mitochondrial energetic status. Melser S, Chatelain EH, Lavie J, Mahfouf W, Jose C, Obre E, Goorden S, Priault M, Elgersma Y, Rezvani HR, Rossignol R, Bénard G. |
| ***RNF185*** | ring finger protein 185 | Mitophagy core | PLoS One. 2011;6(9):e24367.  RNF185, a novel mitochondrial ubiquitin E3 ligase, regulates autophagy through interaction with BNIP1. Tang F, Wang B, Li N, Wu Y, Jia J, Suo T, Chen Q, Liu YJ, Tang J. |
| ***PARK7*** | parkinson protein 7 | Mitophagy core | Trends Biochem Sci. 2015 Apr;40(4):200–210. Mitochondrial dysfunction and mitophagy in Parkinson's: from familial to sporadic disease. Ryan BJ, Hoek S, Fon EA, Wade-Martins R. |
| ***PARK2*** | parkinson protein 2, E3 ubiquitin protein ligase (parkin) | Mitophagy core | J Cell Biol. 2008 Dec 1;183(5):795-803.  Parkin is recruited selectively to impaired mitochondria and promotes their autophagy. Narendra D, Tanaka A, Suen DF, Youle RJ. |
| ***TGM2*** | transglutaminase 2 | Mitophagy core | Cell Death Differ. 2015 Mar;22(3):408-18.  Transglutaminase 2 ablation leads to mitophagy impairment associated with a metabolic shift towards aerobic glycolysis. Rossin F, D'Eletto M, Falasca L, Sepe S, Cocco S, Fimia GM, Campanella M, Mastroberardino PG, Farrace MG, Piacentini M. |
| ***BCL2L13*** | BCL2-like 13 (apoptosis facilitator) | Mitophagy core | Autophagy. 2015;11(10):1932-3.  BCL2L13 is a mammalian homolog of the yeast mitophagy receptor Atg32. Otsu K, Murakawa T, Yamaguchi O. |
| ***HSPA1L*** | Heat Shock Protein Family A (Hsp70) Member 1 Like | Mitophagy core | Redox Biol. 2015;4:6–13. Mitochondrial dynamics and mitochondrial quality control. Ni HM, Williams JA, Ding WX. |
| ***BAG4*** | Bcl2-associated athanogene 4 (BAG4, | Mitophagy core | Redox Biol. 2015;4:6–13. Mitochondrial dynamics and mitochondrial quality control. Ni HM, Williams JA, Ding WX. |
| ***TOMM7*** | Translocase Of Outer Mitochondrial Membrane 7 | Mitophagy core | Redox Biol. 2015;4:6–13. Mitochondrial dynamics and mitochondrial quality control. Ni HM, Williams JA, Ding WX. |
| ***AMBRA1*** | autophagy/beclin-1 regulator 1 | Mitophagy core | J Neurosci. 2011 Jul 13;31(28):10249-61.  Parkin interacts with Ambra1 to induce mitophagy. Van Humbeeck C1, Cornelissen T, Hofkens H, Mandemakers W, Gevaert K, De Strooper B, Vandenberghe W. |
| ***BECN1*** | beclin 1, autophagy related | Mitophagy core | Hum Mol Genet. 2011 Jun 1;20(11):2091–2102. Parkin mediates beclin-dependent autophagic clearance of defective mitochondria and ubiquitinated Abeta in AD models.  Khandelwal PJ, Herman AM, Hoe HS, Rebeck GW, Moussa CE. |
| ***UBE2D2*** | ubiquitin-conjugating enzyme E2D 2 | Mitophagy core | J Cell Sci. 2014 Aug 1;127(Pt 15):3280-93. The ubiquitin-conjugating enzymes UBE2N, UBE2L3 and UBE2D2/3 are essential for Parkin-dependent mitophagy. Geisler S, Vollmer S, Golombek S, Kahle PJ. |
| ***UBE2L3*** | ubiquitin-conjugating enzyme E2L 3 | Mitophagy core | J Cell Sci. 2014 Aug 1;127(Pt 15):3280-93. The ubiquitin-conjugating enzymes UBE2N, UBE2L3 and UBE2D2/3 are essential for Parkin-dependent mitophagy. Geisler S, Vollmer S, Golombek S, Kahle PJ. |
| ***UBE2D3*** | ubiquitin-conjugating enzyme E2D 3 | Mitophagy core | J Cell Sci. 2014 Aug 1;127(Pt 15):3280-93. The ubiquitin-conjugating enzymes UBE2N, UBE2L3 and UBE2D2/3 are essential for Parkin-dependent mitophagy. Geisler S, Vollmer S, Golombek S, Kahle PJ. |
| ***UBE2N*** | ubiquitin-conjugating enzyme E2N | Mitophagy core | J Cell Sci. 2014 Aug 1;127(Pt 15):3280-93. The ubiquitin-conjugating enzymes UBE2N, UBE2L3 and UBE2D2/3 are essential for Parkin-dependent mitophagy. Geisler S, Vollmer S, Golombek S, Kahle PJ. |
| ***RAB5A*** | RAB5A, member RAS oncogene family | Mitophagy core | Nat Commun. 2017 Jan 30;8:14050. A Rab5 endosomal pathway mediates Parkin-dependent mitochondrial clearance. Hammerling BC, Najor RH, Cortez MQ, Shires SE, Leon LJ, Gonzalez ER, Boassa D, Phan S, Thor A, Jimenez RE, Li H, Kitsis RN, Dorn GW II, Sadoshima J, Ellisman MH, Gustafsson ÅB. |
| ***RAB5B*** | RAB5B, member RAS oncogene family | Mitophagy core | Nat Commun. 2017 Jan 30;8:14050. A Rab5 endosomal pathway mediates Parkin-dependent mitochondrial clearance. Hammerling BC, Najor RH, Cortez MQ, Shires SE, Leon LJ, Gonzalez ER, Boassa D, Phan S, Thor A, Jimenez RE, Li H, Kitsis RN, Dorn GW II, Sadoshima J, Ellisman MH, Gustafsson ÅB. |
| ***RAB5C*** | RAB5C, member RAS oncogene family | Mitophagy core | Nat Commun. 2017 Jan 30;8:14050. A Rab5 endosomal pathway mediates Parkin-dependent mitochondrial clearance. Hammerling BC, Najor RH, Cortez MQ, Shires SE, Leon LJ, Gonzalez ER, Boassa D, Phan S, Thor A, Jimenez RE, Li H, Kitsis RN, Dorn GW II, Sadoshima J, Ellisman MH, Gustafsson ÅB. |
| ***RAB7A*** | Ras-Associated Protein RAB7 | Mitophagy core | Sci Adv. 2018 Nov 21;4(11):eaav0443. RAB7A phosphorylation by TBK1 promotes mitophagy via the PINK-PARKIN pathway. Heo JM, Ordureau A, Swarup S, Paulo JA, Shen K, Sabatini DM, Harper JW. |
| ***FLCN*** | Folliculin | Mitophagy core | Sci Adv. 2018 Nov 21;4(11):eaav0443. RAB7A phosphorylation by TBK1 promotes mitophagy via the PINK-PARKIN pathway. Heo JM, Ordureau A, Swarup S, Paulo JA, Shen K, Sabatini DM, Harper JW. |
| ***FNIP1*** | Folliculin Interacting Protein 1 | Mitophagy core | Sci Adv. 2018 Nov 21;4(11):eaav0443. RAB7A phosphorylation by TBK1 promotes mitophagy via the PINK-PARKIN pathway. Heo JM, Ordureau A, Swarup S, Paulo JA, Shen K, Sabatini DM, Harper JW. |
| ***NIPSNAP1*** | Nipsnap Homolog 1 | Mitophagy core | Dev Cell. 2019 May 20;49(4):509-525.e12.  NIPSNAP1 and NIPSNAP2 Act as .Eat Me. Signals for Mitophagy. Princely Abudu Y, Pankiv S, Mathai BJ, Håkon Lystad A, Bindesbøll C, Brenne HB, Yoke Wui Ng M, Thiede B, Yamamoto A, Mutugi Nthiga T, Lamark T, Esguerra CV, Johansen T, Simonsen A. |
| ***NIPSNAP2*** | Nipsnap Homolog 2 | Mitophagy core | Dev Cell. 2019 May 20;49(4):509-525.e12.  NIPSNAP1 and NIPSNAP2 Act as .Eat Me. Signals for Mitophagy. Princely Abudu Y, Pankiv S, Mathai BJ, Håkon Lystad A, Bindesbøll C, Brenne HB, Yoke Wui Ng M, Thiede B, Yamamoto A, Mutugi Nthiga T, Lamark T, Esguerra CV, Johansen T, Simonsen A. |
| ***SESN2*** | sestrin 2 | Mitophagy core | Sci Rep. 2018 Jan 12;8(1):615.  SESN2 facilitates mitophagy by helping Parkin translocation through ULK1 mediated Beclin1 phosphorylation. Kumar A, Shaha C. |
| ***RB1CC1*** | RB1-inducible coiled-coil 1 | Mitophagy core | Mol Cell. 2019 Apr 18;74(2):347-362.e6.  Spatiotemporal Control of ULK1 Activation by NDP52 and TBK1 during Selective Autophagy. Vargas JNS, Wang C, Bunker E, Hao L, Maric D, Schiavo G, Randow F, Youle RJ. |
| ***ULK1*** | unc-51 like autophagy activating kinase 1 | Mitophagy core | Mol Cell. 2019 Apr 18;74(2):347-362.e6.  Spatiotemporal Control of ULK1 Activation by NDP52 and TBK1 during Selective Autophagy. Vargas JNS, Wang C, Bunker E, Hao L, Maric D, Schiavo G, Randow F, Youle RJ. |
| ***SQSTM1*** | sequestosome 1 | Mitophagy-specific cargo receptors | Nat Cell Biol. 2010 Feb;12(2):119-31.  PINK1/Parkin-mediated mitophagy is dependent on VDAC1 and p62/SQSTM1. Geisler S, Holmström KM, Skujat D, Fiesel FC, Rothfuss OC, Kahle PJ, Springer W. |
| ***BNIP3L*** | BCL2/adenovirus E1B 19kDa interacting protein 3-like | Mitophagy-specific cargo receptors | Mol Cell. 2014 Jan 23;53(2):167-78.  Interactions between autophagy receptors and ubiquitin-like proteins form the molecular basis for selective autophagy. Rogov V, Dötsch V, Johansen T, Kirkin V. |
| ***FUNDC1*** | FUN14 domain containing 1 | Mitophagy-specific cargo receptors | Mol Cell. 2014 Jan 23;53(2):167-78.  Interactions between autophagy receptors and ubiquitin-like proteins form the molecular basis for selective autophagy. Rogov V, Dötsch V, Johansen T, Kirkin V. |
| ***OPTN*** | optineurin | Mitophagy-specific cargo receptors | Proc Natl Acad Sci U S A. 2016 Apr 12;113(15):4039-44.  Phosphorylation of OPTN by TBK1 enhances its binding to Ub chains and promotes selective autophagy of damaged mitochondria. Richter B, Sliter DA, Herhaus L, Stolz A, Wang C, Beli P, Zaffagnini G, Wild P, Martens S, Wagner SA, Youle RJ, Dikic I. |
| ***CALCOCO2*** | calcium binding and coiled-coil domain 2 | Mitophagy-specific cargo receptors | Nature. 2015 Aug 20;524(7565):309-314.  The ubiquitin kinase PINK1 recruits autophagy receptors to induce mitophagy. Lazarou M, Sliter DA, Kane LA, Sarraf SA, Wang C, Burman JL, Sideris DP, Fogel AI, Youle RJ. |
| ***PHB*** | prohibitin | Mitophagy-specific cargo receptors | Cell. 2017 Jan 12;168(1-2):224-238.e10.  Prohibitin 2 Is an Inner Mitochondrial Membrane Mitophagy Receptor. Wei Y, Chiang WC, Sumpter R Jr, Mishra P, Levine B. |
| ***PHB2*** | prohibitin 2 | Mitophagy-specific cargo receptors | Cell. 2017 Jan 12;168(1-2):224-238.e10.  Prohibitin 2 Is an Inner Mitochondrial Membrane Mitophagy Receptor. Wei Y, Chiang WC, Sumpter R Jr, Mishra P, Levine B. |
| ***TAX1BP1*** | Tax1 (human T-cell leukemia virus type I) binding protein 1 | Mitophagy-specific cargo receptors | Nature. 2015 Aug 20;524(7565):309-314.  The ubiquitin kinase PINK1 recruits autophagy receptors to induce mitophagy. Lazarou M, Sliter DA, Kane LA, Sarraf SA, Wang C, Burman JL, Sideris DP, Fogel AI, Youle RJ. |
| ***LRPPRC*** | leucine-rich pentatricopeptide repeat containing | Negative regulator of Mitophagy | PLoS One. 2014 Apr 10;9(4):e94903.  Autophagy inhibitor LRPPRC suppresses mitophagy through interaction with mitophagy initiator Parkin. Zou J, Yue F, Li W, Song K, Jiang X, Yi J, Liu L. |
| ***USP30*** | ubiquitin specific peptidase 30 | Negative regulator of Mitophagy | Nature. 2014 Jun 19;510(7505):370-5.  The mitochondrial deubiquitinase USP30 opposes parkin-mediated mitophagy. Bingol B, Tea JS, Phu L, Reichelt M, Bakalarski CE, Song Q, Foreman O, Kirkpatrick DS, Sheng M. |
| ***USP35*** | ubiquitin specific peptidase 35 | Negative regulator of Mitophagy | Autophagy. 2015 Apr 3;11(4):595–606. Deubiquitinating enzymes regulate PARK2-mediated mitophagy.  Wang Y, Serricchio M, Jauregui M, Shanbhag R, Stoltz T, Di Paolo CT, Kim PK, McQuibban GA. |
| ***USP15*** | ubiquitin specific peptidase 15 | Negative regulator of Mitophagy | Hum Mol Genet. 2014 Oct 1;23(19):5227-42.  The deubiquitinase USP15 antagonizes Parkin-mediated mitochondrial ubiquitination and mitophagy. Cornelissen T, Haddad D, Wauters F, Van Humbeeck C, Mandemakers W, Koentjoro B, Sue C, Gevaert K, De Strooper B, Verstreken P, Vandenberghe W. |
| ***TMEM173*** | Transmembrane Protein 173 | Negative regulator of Mitophagy | Cell Signal. 2017 Jul;35:73-83.  MITA modulated autophagy flux promotes cell death in breast cancer cells. Bhatelia K, Singh K, Prajapati P, Sripada L, Roy M, Singh R. |
| ***SIAH3*** | Siah E3 Ubiquitin Protein Ligase Family Member 3 | Negative regulator of Mitophagy | Redox Biol. 2015;4:6–13. Mitochondrial dynamics and mitochondrial quality control. Ni HM, Williams JA, Ding WX. |
| ***BCL2L1*** | BCL2-like 1 | Negative regulator of Mitophagy | Mol Cell. 2014 Aug 7;55(3):451-66.  Bcl-2 family proteins participate in mitochondrial quality control by regulating Parkin/PINK1-dependent mitophagy. Hollville E, Carroll RG, Cullen SP, Martin SJ. |
| ***MCL1*** | Myeloid Cell Leukemia 1 | Negative regulator of Mitophagy | Mol Cell. 2014 Aug 7;55(3):451-66.  Bcl-2 family proteins participate in mitochondrial quality control by regulating Parkin/PINK1-dependent mitophagy. Hollville E, Carroll RG, Cullen SP, Martin SJ. |
| ***LKKR2*** | Leucine Rich Repeat Kinase 2 | Negative regulator of Mitophagy | Hum Mol Genet. 2019 May 15;28(10):1645-1660.  LRRK2 impairs PINK1/Parkin-dependent mitophagy via its kinase activity: pathologic insights into Parkinson's disease. Bonello F, Hassoun SM, Mouton-Liger F, Shin YS, Muscat A, Tesson C, Lesage S, Beart PM, Brice A, Krupp J, Corvol JC, Corti O. |
| ***ZNF746*** | zinc finger protein 746 | Negative regulator of PGC1alpha | J Neurosci. 2015 Sep 16;35(37):12833–12844. Mitochondrial Quality Control via the PGC1α-TFEB Signaling Pathway Is Compromised by Parkin Q311X Mutation But Independently Restored by Rapamycin. Siddiqui A, Bhaumik D, Chinta SJ, Rane A, Rajagopalan S, Lieu CA, Lithgow GJ, Andersen JK. |
| ***MFN1*** | mitofusin 1 | Fusion | J Cell Biol. 2003 Jan 20;160(2):189-200. Epub 2003 Jan 13. Mitofusins Mfn1 and Mfn2 coordinately regulate mitochondrial fusion and are essential for embryonic development. Chen H, Detmer SA, Ewald AJ, Griffin EE, Fraser SE, Chan DC. |
| ***MFN2*** | mitofusin 2 | Fusion | J Cell Biol. 2003 Jan 20;160(2):189-200. Epub 2003 Jan 13. Mitofusins Mfn1 and Mfn2 coordinately regulate mitochondrial fusion and are essential for embryonic development. Chen H, Detmer SA, Ewald AJ, Griffin EE, Fraser SE, Chan DC. |
| ***OPA1*** | optic atrophy 1 (autosomal dominant) | Fusion | Proc Natl Acad Sci U S A. 2004 Nov 9;101(45):15927-32. OPA1 requires mitofusin 1 to promote mitochondrial fusion. Cipolat S, Martins de Brito O, Dal Zilio B, Scorrano L. |
| ***ROCK1*** | Rho-associated, coiled-coil containing protein kinase 1 | Fission | Cell Metab. 2012 Feb 8;15(2):186-200.  Mitochondrial fission triggered by hyperglycemia is mediated by ROCK1 activation in podocytes and endothelial cells. Wang W, Wang Y, Long J, Wang J, Haudek SB, Overbeek P, Chang BH, Schumacker PT, Danesh FR. |
| ***MIEF1*** | Mitochondrial Elongation Factor 1 | Fission | Mol Biol Cell. 2013 Mar;24(5):659-67.  Fis1, Mff, MiD49, and MiD51 mediate Drp1 recruitment in mitochondrial fission. Losón OC, Song Z, Chen H, Chan DC. |
| ***MIEF2*** | Mitochondrial Elongation Factor 2 | Fission | Mol Biol Cell. 2013 Mar;24(5):659-67.  Fis1, Mff, MiD49, and MiD51 mediate Drp1 recruitment in mitochondrial fission. Losón OC, Song Z, Chen H, Chan DC. |
| ***DNM1L*** | dynamin 1-like | Fission | J Cell Biol. 2016 Feb 15;212(4):379–387. Metabolic regulation of mitochondrial dynamics. Mishra P, Chan DC. |
| ***FIS1*** | Fission, Mitochondrial 1 | Fission | Mol Biol Cell. 2013 Mar;24(5):659-67.  Fis1, Mff, MiD49, and MiD51 mediate Drp1 recruitment in mitochondrial fission. Losón OC, Song Z, Chen H, Chan DC. |
| ***HTRA2*** | HtrA serine peptidase 2 | Fission | Exp Cell Res. 2010 Apr 15;316(7):1213-24.  Modulation of mitochondrial function and morphology by interaction of Omi/HtrA2 with the mitochondrial fusion factor OPA1. Kieper N, Holmström KM, Ciceri D, Fiesel FC, Wolburg H, Ziviani E, Whitworth AJ, Martins LM, Kahle PJ, Krüger R. |
| ***MFF*** | mitochondrial fission factor | Fission | Mol Biol Cell. 2013 Mar;24(5):659-67.  Fis1, Mff, MiD49, and MiD51 mediate Drp1 recruitment in mitochondrial fission. Losón OC, Song Z, Chen H, Chan DC. |
| ***SIRT2*** | sirtuin 2 | Transcription Factors involved in Mitophagy | 1)Biochem Biophys Res Commun. 2017 Jul 8;488(4):603-608.  Sirtuin inhibition leads to autophagy and apoptosis in porcine preimplantation blastocysts. Kim MG; 2) Mol Neurobiol. 2017 Aug;54(6):4021-4040.  Mitochondrial Metabolism Power SIRT2-Dependent Deficient Traffic Causing Alzheimer's-Disease Related Pathology. Silva DF |
| ***SIRT3*** | sirtuin 3 | Transcription Factors involved in Mitophagy | Biochim Biophys Acta. 2017 Aug;1863(8):1973-1983.  Sirt3 deficiency exacerbates diabetic cardiac dysfunction: Role of Foxo3A-Parkin-mediated mitophagy. Yu W |
| ***SIRT5*** | sirtuin 5 | Transcription Factors involved in Mitophagy | Autophagy. 2015;11(2):253-70.  SIRT5 regulation of ammonia-induced autophagy and mitophagy. Polletta L |
| ***SOX2*** | SRY-box 2 | Transcription Factors involved in Mitophagy | Autophagy. 2017 Jul 19:0. BNIP3L-dependent Mitophagy Accounts for Mitochondrial Clearance during Three Factors Induced Somatic Cell Reprogramming. Xiang G |
| ***MITF*** | melanogenesis associated transcription factor | Transcription Factors involved in Mitophagy | J Cell Biol. 2015 Aug 3;210(3):435-50. MiT/TFE transcription factors are activated during mitophagy downstream of Parkin and Atg5. Nezich CL, Wang C, Fogel AI, Youle RJ. |
| ***TFE3*** | transcription factor binding to IGHM enhancer 3 [*Homo sapiens* | Transcription Factors involved in Mitophagy | J Cell Biol. 2015 Aug 3;210(3):435-50. MiT/TFE transcription factors are activated during mitophagy downstream of Parkin and Atg5. Nezich CL, Wang C, Fogel AI, Youle RJ. |
| ***TFEB*** | transcription factor EB | Transcription Factors involved in Mitophagy | J Cell Biol. 2015 Aug 3;210(3):435-50. MiT/TFE transcription factors are activated during mitophagy downstream of Parkin and Atg5. Nezich CL, Wang C, Fogel AI, Youle RJ. |
| ***TFEC*** | transcription factor EC | Transcription Factors involved in Mitophagy | Pharmacol Res. 2015 Sep;99:36-43.  The MITF family of transcription factors: Role in endolysosomal biogenesis, Wnt signaling, and oncogenesis. Ploper D |
| ***SREBF1*** | sterol regulatory element binding transcription factor 1 | Transcription Factors involved in Mitophagy | Autophagy. 2014 Aug;10(8):1476-7. SREBF1 links lipogenesis to mitophagy and sporadic Parkinson disease. Ivatt RM |
| ***SREBF2*** | sterol regulatory element binding transcription factor 2 | Transcription Factors involved in Mitophagy | Int J Biochem Cell Biol. 2014 Oct;55:196-208.  Leishmania donovani activates SREBP2 to modulate macrophage membrane cholesterol and mitochondrial oxidants for establishment of infection. Mukherjee M |

**Additional file 1: Table S6. Phenotype and clinical features of the post-mortem ADNP patient carrying the c.1676duplA/p.His559Glnfs*3 mutation in comparison with a cohort study by Van Dijck et al. (2019) entailing 78 HVDAS individuals.**

| **Clinical features** | **Post-mortem patient**  **(p.His559Glnfs*3)** | **Van Dijck et al. (2019)**  **Cohort study** |
| --- | --- | --- |
| ***General information*** | | |
| Gender | Male | 34 females and 44 males |
| Duration gestation (weeks) | 32 | 38.7 |
| Birth weight (g) | 1970 | 3065.2 |
| Birth length (cm) | 42 | 49.2 |
| Weight at last observation (g) | 20500 | Not defined |
| Height at last observation (cm) | 113 | Not defined |
| ***Delay*** | | |
| Growth retardation / short stature | No | In 23.2% of patients |
| Intellectual disability | Moderate | 12.3% mild  35.6% moderate  52.1% severe |
| Age sitting up independently | 9 months | 1.1 years |
| Delayed walking independently | Yes | 86.8% of patients |
| Age walking independently | 18 months | 2.5 years |
| Speech delay | Yes | 98.6% of patients |
| Age at first words | Not defined | 2.5 years |
| Bladder training delay | Yes | 81.1% of patients |
| ***Feeding problems*** | | |
| Gastrointestinal problems | yes | 83.3% of patients |
| ***Neurological problems and behavior*** | | |
| Autism spectrum disorder | Yes | 92.8% of patients |
| ADHD | No | 43.9% of patients |
| Hypotonia | Yes | 78.3% of patients |
| Hypertonia | n.d. | 3.8% of patients |
| Seizures | Yes | 16.2% of patients |
| Mood disorder | Yes | 56.3% of patients |
| Obsessive-compulsive behavior | No | 64.0% of patients |
| Temper tantrums/aggression | Yes | 83.3% of patients |
| Social behavior | Avoids other children | Not defined |
| ***Craniofacial features*** | | |
| Prominent forehead | Yes | 65.6% of patients |
| Prominent eyelashes | Yes | 16.7% of patients |
| Downward slant palpebral fissures | Yes | 33.3% of patients |
| Pointed chin | Yes | Not defined |
| Wide nasal bridge | Yes | 50.0% of patients |
| High hairline | No | 50.0% of patients |
| Short nose | No | 49.5% of patients |
| Upturned nasal tip | No | 46.7% of patients |
| Broad philtrum | Yes | 16.1% of patients |
| Everted lower lip | No | 45.5% of patients |
| Long philtrum | Yes | 39.3% of patients |
| Large mouth | Yes | 23.2% of patients |
| Thin upper vermillion | No | 70.3% of patients |
| Thick lower vermillion | Yes | 36.4% of patients |
| Widely spaced teeth | Yes | 34.6% of patients |
| Low set or posteriorly rotated ears | Yes | 14.7% of patients |
| Malformed ears | Yes | 48.5% of patients |
| ***Other*** | | |
| Early teething | Yes | 71.1% of patients |
| Visual problems | Hypertelorism | 73.6% of patients |
| Sleep problems | Yes | 65.2% of patients |
| Insensitivity to pain | Yes | 63.6% of patients |
| Small genitalia | Yes | 5.4% of patients |
| Umbilical/inguinal hernia | Umbilical hernia in infancy | 8.5% of patients |
