# Supplementary material for: ADNP dysregulates methylation and mitochondrial gene expression in the cerebellum of a Helsmoortel–Van der Aa syndrome autopsy case
Source: Acta Neuropathol Commun. 2024 Apr 18;12:62. doi: 10.1186/s40478-024-01743-w (PMC11027339; doi:10.1186/s40478-024-01743-w)
Supplement: Supplementary file 7 — Additional file 7: Correlation heatmap of the label-free quantification mass spectrometry (LFQ-MS) experiment in the ADNP cerebellum. Pairwise correlations of protein abundances characterizing the relationships between proteins of the global mass spectrometry experiment. Correlations between all proteins, clustering of protein groups and similarly behaving proteins are represented in the protein-protein correlation matrix calculated on the logarithmic intensities. A strong correlation between technical replicates (n = 5) was observed (red color), indicating high reproducibility as tested by the Pearson correlation. Negative correlations are represented in a blue color (see scaled bar legend). [file 40478_2024_1743_MOESM7_ESM.pdf]

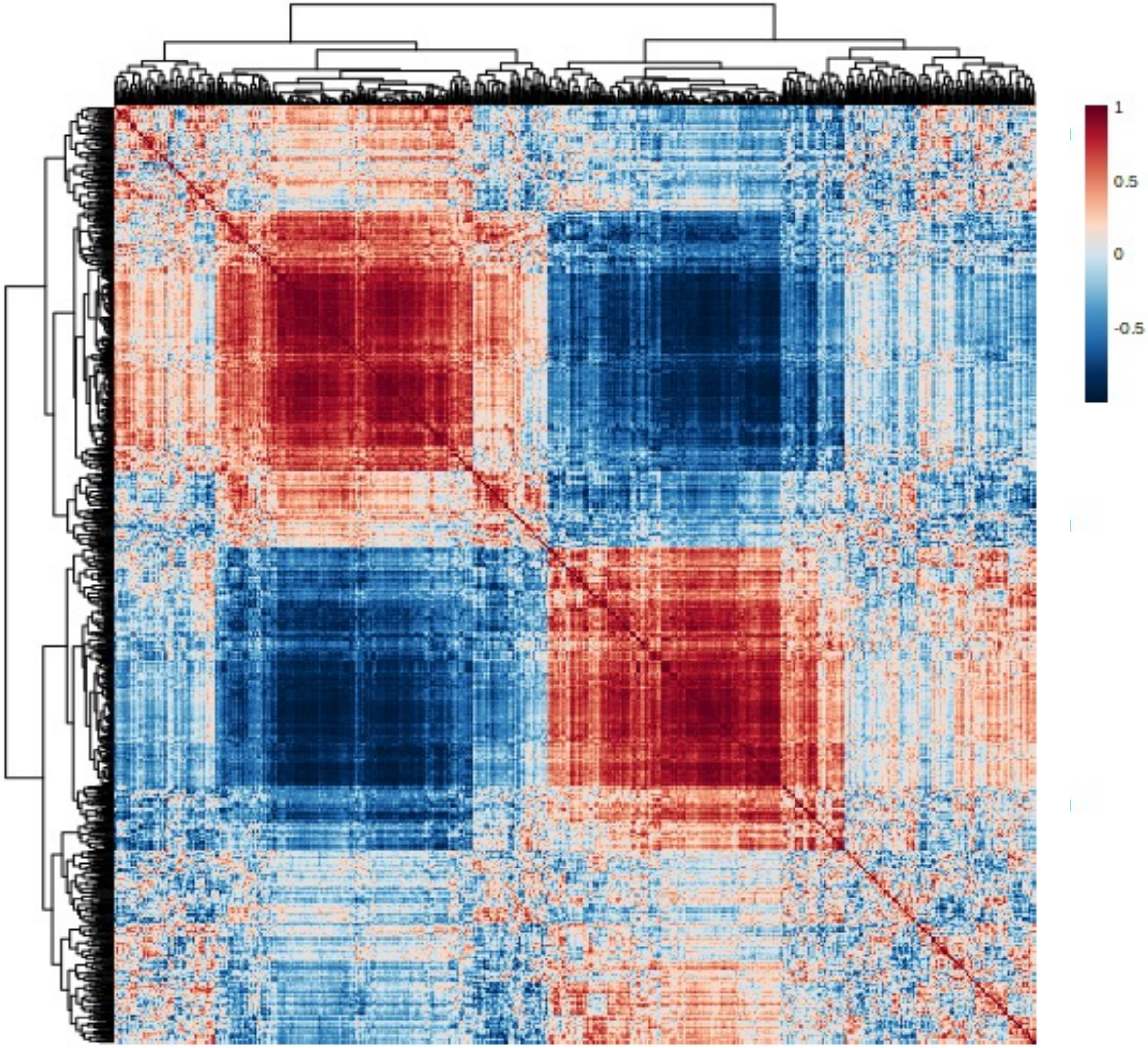


**Supplementary figure S7. Correlation heatmap of the LFQ-MS experiment**. Pairwise correlations of protein abundances characterizing the relationships between proteins of the global mass spectrometry experiment. Correlations between all proteins, clustering of protein groups and similarly behaving proteins are represented in this protein-protein correlation matrix calculated on the logarithmic intensities. A strong correlation between the technical replicates (n =5) was observed (red colour) indicating high reproducibility as tested by the Pearson correlation. Negative correlations are represented in a blue color (see legend).
