# Supplementary figures and images for: ADNP dysregulates methylation and mitochondrial gene expression in the cerebellum of a Helsmoortel–Van der Aa syndrome autopsy case
Source: Acta Neuropathol Commun. 2024 Apr 18;12:62. doi: 10.1186/s40478-024-01743-w (PMC11027339; doi:10.1186/s40478-024-01743-w)

-1.52 1.24

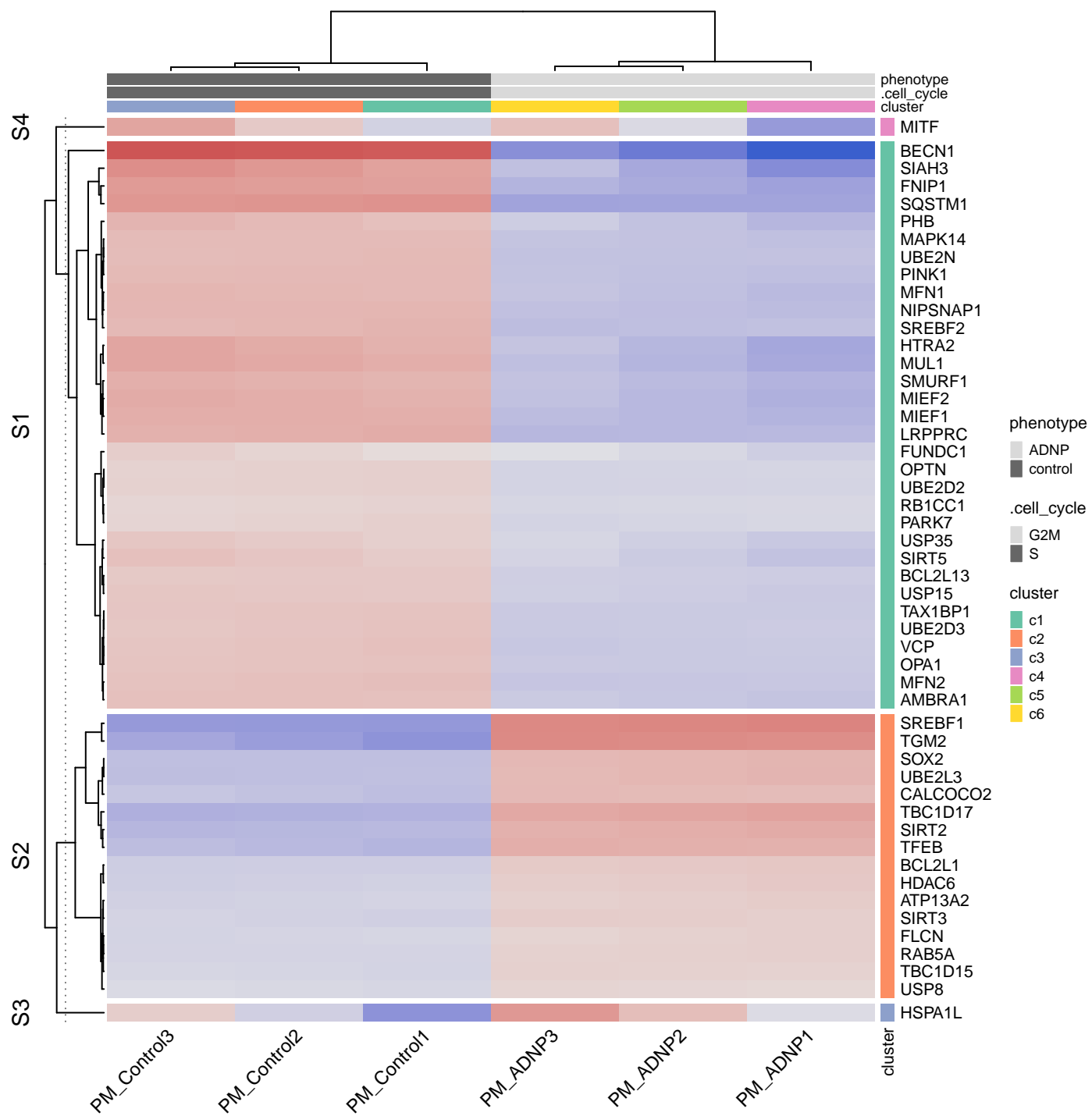

Supplement: Supplementary file 9 — Additional file 9: Mitophagy transcriptomic gene signature in the ADNP cerebellum. Expression levels of mitophagy-related genes in the ADNP patient cerebellum compared to an age-matched control subject. mRNA sequencing demonstrated both an upregulated (red) as well as a downregulated (blue) mitophagy gene signature in the ADNP patient cerebellum as compared to an age-matched control subject. [file 40478_2024_1743_MOESM9_ESM.pdf]
