# Supplementary material for: ADNP dysregulates methylation and mitochondrial gene expression in the cerebellum of a Helsmoortel–Van der Aa syndrome autopsy case
Source: Acta Neuropathol Commun. 2024 Apr 18;12:62. doi: 10.1186/s40478-024-01743-w (PMC11027339; doi:10.1186/s40478-024-01743-w)
Supplement: Supplementary file 10 — Additional file 10: Autophagic flux assessment in ADNP patient-derived lymphoblastoid cell lines using Bafilomycin A1. A. The autophagic flux was determined in ADNP patient-derived and age- and sex-matched control lymphoblastoid cell lines by treatment with 160 nM of Bafilomycin A1 (BAF) for two hours. Protein extracts of untreated and BAF-treated cells were subjected to western blotting using anti-p62/SQSTM1 and anti-LC3 antibodies to assess the autophagic flux. Although p62 expression increased after BAF treatment (+BAF) compared to untreated (-BAF) cells, the difference was not significant in patients (PAT) and controls (CTR). However, the expression of LC3 significantly increased after BAF treatment and was significantly increased in PAT versus CTR post-treatment compared to untreated patient cells (PAT-BAF). All western blots were controlled by GAPDH to ensure equal loading. Image quantification was performed using ImageJ software. B. Graphical representation was performed in GraphPad Prism version 9.3.1 using a 2-way ANOVA with Sidak’s multiple comparisons test to assess the interaction of the genotype (PAT versus CTR) and treatment (-BAF versus +BAF). [file 40478_2024_1743_MOESM10_ESM.pptx]

## Slide 1
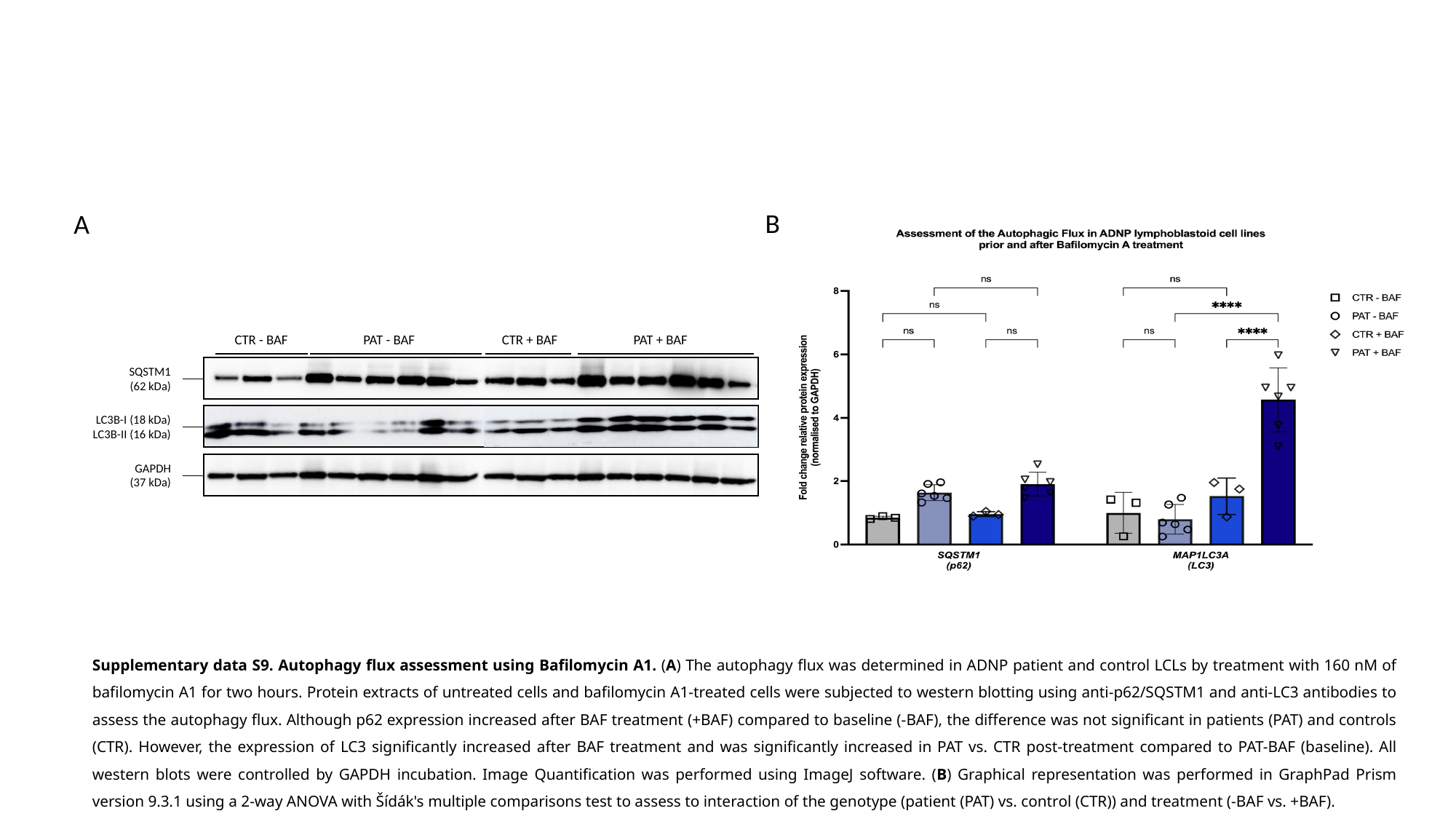

B
A
PAT - BAF
CTR + BAF
PAT + BAF
CTR - BAF
SQSTM1
(62 kDa)
LC3B-I (18 kDa)
LC3B-II (16 kDa)
GAPDH
(37 kDa)
Supplementary data S9. Autophagy flux assessment using Bafilomycin A1. (A) The autophagy flux was determined in ADNP patient and control LCLs by treatment with 160 nM of bafilomycin A1 for two hours. Protein extracts of untreated cells and bafilomycin A1-treated cells were subjected to western blotting using anti-p62/SQSTM1 and anti-LC3 antibodies to assess the autophagy flux. Although p62 expression increased after BAF treatment (+BAF) compared to baseline (-BAF), the difference was not significant in patients (PAT) and controls (CTR). However, the expression of LC3 significantly increased after BAF treatment and was significantly increased in PAT vs. CTR post-treatment compared to PAT-BAF (baseline). All western blots were controlled by GAPDH incubation. Image Quantification was performed using ImageJ software. (B) Graphical representation was performed in GraphPad Prism version 9.3.1 using a 2-way ANOVA with Šídák's multiple comparisons test to assess to interaction of the genotype (patient (PAT) vs. control (CTR)) and treatment (-BAF vs. +BAF).
